# Intermolecular Interactions, Solute Descriptors, and Partition Properties of Neutral Per- and Polyfluoroalkyl Substances (PFAS)

**DOI:** 10.1021/acs.est.3c07503

**Published:** 2023-11-01

**Authors:** Satoshi Endo

**Affiliations:** Health and Environmental Risk Division, National Institute for Environmental Studies (NIES), Onogawa 16-2, Tsukuba 305-8506, Ibaraki, Japan

**Keywords:** fluorotelomer substance, perfluoroalkane sulfonamide, gas chromatographic retention factor, linear solvation energy relationship, hydrogen bonding, chemical partitioning space

## Abstract

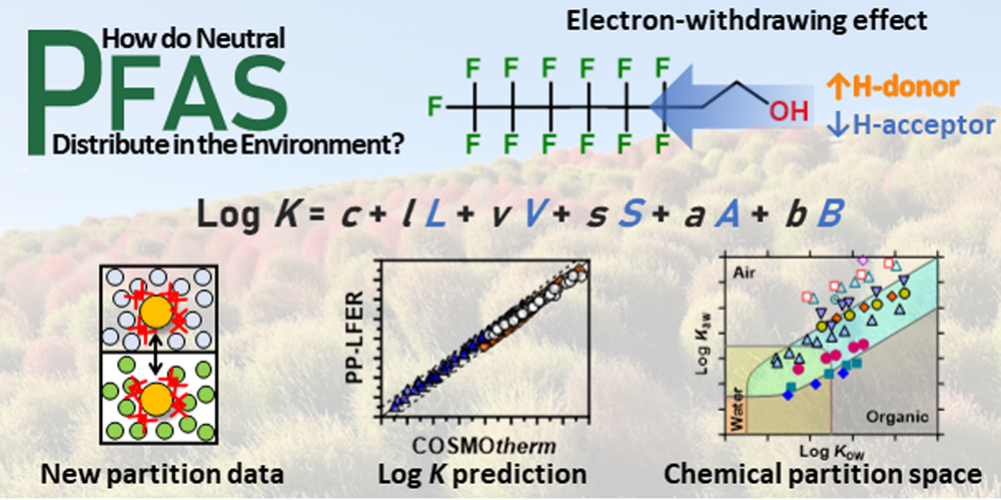

The environmental partition properties of perfluoroalkyl and polyfluoroalkyl substances (PFAS) must be understood for their transport and fate analysis. In this study, isothermal gas chromatographic (GC) retention times of 60 neutral PFAS were measured using four columns with different stationary phase polarities, which indicated varying polar interactions exerted by these substances. The GC data were combined with new octanol/water partition coefficient data from this study and existing partition coefficient data from the literature and used to determine the polyparameter linear free energy relationship (PP-LFER) solute descriptors. A complete set of the solute descriptors was obtained for 47 PFAS, demonstrating the characteristic intermolecular interaction properties, such as hydrogen bonding capabilities influenced by the electron-withdrawing perfluoroalkyl group. The partition coefficients between octanol and water, air and water, and octanol and air predicted by the PP-LFER models agreed with those predicted by the quantum chemically based model COSMO*therm*, suggesting that both models are highly accurate for neutral PFAS and can fill the current large data gaps in partition property data. A chemical partitioning space plot was generated by using the PP-LFER-predicted partition coefficients, showing the primary importance of the air phase for the environmental distribution of nonpolar and weakly polar PFAS and the increasing significance of organic phases with increasing PFAS polarity.

## Abstract 2

Polyparameter linear free energy relationship (PP-LFER) descriptors were determined for 47 neutral PFAS, allowing for the prediction of their partition coefficients.

## Introduction

Per- and polyfluoroalkyl substances (PFAS) are of increasing concern due to their potential adverse effects on human health and ecosystems. Despite the high level of concern, the equilibrium partition properties of PFAS remain to be elucidated. Even basic property data such as octanol/water partition coefficients (*K*_ow_) are scarce for PFAS.^1−3^ To the best of my knowledge, only one scientific paper^4^ has reported measured *K*_ow_ values for neutral PFAS with perfluoroalkyl chain lengths of 4 or more. Similarly, reliable air/water partition coefficients (*K*_aw_) were available for only a few neutral PFAS with (CF_2_)_≥4_ until our group recently reported experimental values for 21 PFAS.^5^

The equilibrium partition of organic compounds is determined by the intermolecular interactions with the surrounding phases, including polar (e.g., hydrogen (H)-bonding) and nonpolar (i.e., van der Waals) interactions. This study focuses on neutral compounds and neutral species of ionizable compounds, since the partitioning of ionized compounds is controlled by different mechanisms.^6^ For neutral organic compounds, polyparameter linear free energy relationships (PP-LFERs) with Abraham’s solute descriptors can quantify the energetic contributions of the relevant intermolecular interactions and describe partition coefficients in general.^7^ The PP-LFER equation most applicable to PFAS is^8,9^
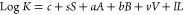
1where *K* is the partition coefficient (also referred to as the partition constant or the partition ratio) between the two phases of interest. The uppercase letters on the right side of eq 1 are the solute descriptors: *S*, dipolarity/polarizability parameter; *A*, solute H-bond donor property; *B*, solute H-bond acceptor property; *V*, McGowan’s molar volume; and *L*, the logarithmic hexadecane/air partition coefficient (*K*_Hxd/air_) at 25 °C. The lowercase letters are referred to as system parameters, are calibrated by least-squares multiple linear regression (MLR) with typically 50 or more calibration data, and reflect the complementary interaction properties of the two phases. The terms with *S*, *A*, and *B* describe the polar interactions, while those with *V* and *L* describe the nonspecific cavity formation and nonpolar interactions.

The PP-LFERs can conveniently predict a variety of partition coefficients for a given compound. While solute descriptors are available for several thousand compounds,^10^ they are rarely available for PFAS. In the past, Goss et al.^11^ determined solute descriptors for four fluorotelomer alcohols (FTOHs) and four fluorotelomer olefins (FTOs). Later, Endo and Goss^9^ recalibrated the solute descriptors for FTOHs, which were evaluated again by Abraham and Acree.^12^ In addition, descriptors for very short PFAS (C_≤3_, mostly refrigerants) have been reported,^13,14^ and the ABSOLV database stored on the UFZ-LSER website^10^ lists descriptor values for several per- and polyfluorinated alkanes and alcohols of unknown origin. Solute descriptors are unavailable for other PFAS, including those that have been frequently detected in the environment such as other fluorotelomer compounds and perfluoroalkanesulfonamides (PFASAs) with different substitution patterns.^15−17^

In a previous study, our group measured the values of *K*_Hxd/air_, the decadic logarithm of which is *L* in eq 1, for 64 neutral PFAS to elucidate their nonpolar interaction properties.^18^ The study showed that *L* can be explained by summing the contributions from the fluorinated and nonfluorinated parts of the molecule. For the polar interaction properties, however, such a simple additive principle may not work because the polar functional group can be significantly influenced by the strongly electron-withdrawing perfluoroalkyl group. In fact, previous studies have shown that *A* values for 4:2, 6:2, and 8:2 FTOHs are higher than those of nonfluorinated alkyl alcohols and that *B* has the opposite relationship.^9,11,12^ Such effects should have a major impact on the partition properties of fluorinated organic compounds in general.

The purpose of this study was to characterize the interaction properties of neutral PFAS and enable the prediction of their partition coefficients by determining the PP-LFER solute descriptors. While the most recent definition of PFAS includes even compounds with only one perfluorinated C atom,^19^ the focus of this study is on PFAS with (CF_2_)_≥4_, as only highly fluorinated PFAS show characteristic partition properties compared to non-PFAS.^20^ Isothermal gas chromatography (GC) retention times were measured on capillary columns of differing polarity. GC retention data are useful because they are relatively easy and fast to measure and are equivalent to partition coefficients, reflecting the intermolecular interactions between the compound and stationary phase. The measured GC retention data were combined with new *K*_ow_ data from this study and existing partition coefficient data from the literature and used to calibrate the PFAS solute descriptors. The resulting descriptors were used to predict partition coefficients, which were compared to predictions by the quantum chemically based COSMO*therm* model for validation. Finally, the PP-LFER-predicted partition coefficients were used to create a chemical partitioning space plot to graphically compare the environmental partition characteristics of various PFAS and non-PFAS.

## Materials and Methods

### Chemicals

Sixty PFAS used in this study are listed in Table 1. Table S1 in the Supporting Information includes the providers, purities, and CAS registry numbers. Of these 60, 59 were used in our previous study^18^ and 10:2 FTO was added to the current list. In addition, 78 nonfluorinated reference compounds with known solute descriptors were analyzed for GC retention times. A complete list of these compounds with suppliers, purities, and CAS Registry numbers is provided in Table S2. GC grade acetone and *n*-hexane and LC/MS grade methanol from Fujifilm Wako Pure Chemical Corporation (Osaka, Japan) and *n*-octan-1-ol (>99.5%) from Tokyo Chemical Industry (Tokyo, Japan) were used as solvents. Tap water was treated by reverse osmosis and further purified with an Ultrapure Water System (RFU665DA, Advantec, Tokyo, Japan).

**Table 1:**
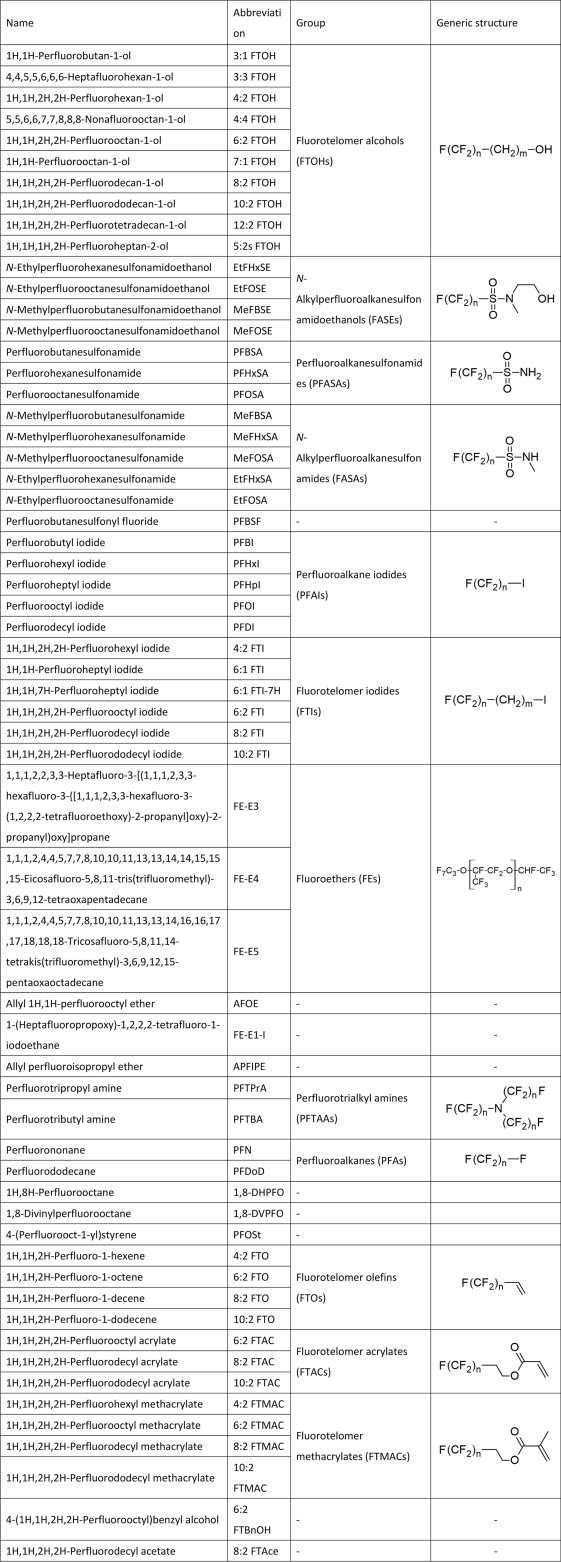
List of PFAS Used in This Study

### Isothermal GC Retention Time Measurement

The GC columns used were, from low to high polarity, HP-5ms Ultra Inert (poly[5% phenyl/95% methyl]siloxane equivalent; Agilent Technologies, Santa Clara, CA), DB-200 (poly[35% trifluoropropyl/65% methyl]siloxane; Agilent Technologies), DB-225ms (poly[50% cyanopropylphenyl/50% methyl]siloxane equivalent; Agilent Technologies), and SolGel-WAX (polyethylene glycol; Trajan, Ringwood, Australia). Column dimensions were all 30 m × 0.25 mm i.d. with a coating thickness of 0.25 μm.

The method for GC retention time measurements follows that of a previous study.^18^ Briefly, a 7890A/5975C GC/MS instrument (Agilent Technologies) equipped with an MPS2 autosampler and CIS4 injector (Gerstel, Mülheim an der Ruhr, Germany) was used for all measurements. For each column, isothermal retention times were measured at four temperatures in the range of 30–120 °C to cover all target compounds. The temperature at the injector and transfer line was fixed at 120 °C. The carrier gas was He at a flow rate of 1.2 mL/min. Compounds were introduced into the GC by injecting either headspace (100–250 μL) above the pure compound or solution in acetone (1 μL). Split injections were performed with a split ratio of 20–250. Column dead time (*t*_0_) was determined by injecting air and measuring Ar (*m*/*z* 40). From the measured retention time (*t*), the retention factor (*k*) was calculated as *k* = (*t*–*t*_0_)/*t*_0_. Measurements with *t* < *t*_0_ + 0.1 min were considered too short and discarded. For each column and temperature, *k* values were obtained for 29–52 PFAS and 29–48 reference compounds. Only one injection was performed for each compound because repeated measurements showed that the *k* variation was typically within 0.01 log unit. Because multiple isomer peaks were observed in the chromatograms for fluoroether (FE)-F4 and FE-F5, the log *k*′ value corresponding to the middle peak was used to represent all isomers, as previously done.^18^

### *K*_ow_ Measurement

*K*_ow_ was measured for nine PFAS (Table 2) to supplement the data set for descriptor determination. The nine PFAS were selected based on measurement feasibility and structural diversity. The shared-headspace or batch partition method was used as described previously.^5^ Details of the methods are given in SI-1.

**Table 2:** Measured Log *K*_ow_ Values at 25 °C for Neutral PFAS

	Mean ± SD	Method
3:3 FTOH	2.46 ± 0.00	SH
4:4 FTOH	3.54 ± 0.00	SH
7:1 FTOH	5.01 ± 0.02	SH
5:2s FTOH	3.80 ± 0.03	SH
6:1 FTI	5.57 ± 0.01	SH
6:1 FTI-7H	4.90 ± 0.01	SH
PFBSA	2.85 ± 0.02	BP
MeFBSA	3.51 ± 0.00	BP
MeFBSE	3.11 ± 0.04	BP
4:2 FTOH^a^	3.30 ± 0.04	BP
6:2 FTOH^a^	4.54 ± 0.01	BP
8:2 FTOH^a^	5.58 ± 0.06	BP

a Data are from ref (4). SH, shared-headspace method; BP, batch partition method.

### Solute Descriptor Determination

The standard procedure for solute descriptor determination was as follows. First, the system parameters in eq 1 were calibrated for each column (or partition system) and temperature by MLR using log *k* (or log *K*) data and solute descriptors for reference compounds. Solute descriptor values for the reference compounds are listed in Table S3. Dimethylformamide, 1-pentylamine, dimethylsulfoxide, and dodecamethylcyclohexasiloxane (D6) were removed from the log *k* data sets, because they frequently appeared as outliers (>3 SD) of the PP-LFER model fit. Reference data sets for hexadecane/water partition coefficients (*K*_Hxd/w_),^21^*K*_ow_,^22^ and octanol/air partition coefficients (*K*_oa_)^23^ were taken from the cited references and amended with data for organosilicon compounds.^24,25^ Similar to PFAS, organosilicon compounds have weak van der Waals interaction properties^9^ and it may be beneficial to include them in reference data sets. The solute descriptors for PFAS were then back-calculated by using the calibrated PP-LFER equations. Of the five descriptors, *V* can be calculated from the molecular structure^26^ (note: the fragment value for F from Goss et al.^11^ was used), and *L* was available for the PFAS studied.^18^ Thus, the left side of eq 2 was known, and MLR was performed to obtain *S*, *A*, and *B* on the right side.
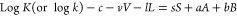
2To determine the three descriptors, a minimum of three *k* and *K* data are required for a given PFAS compound. The data used here were: *k* measured for 60 PFAS in this study, *K*_Hxd/w_ for 21 PFAS,^5^*K*_ow_ for 12 PFAS (see the Results and Discussion section), and *K*_oa_ for 3 PFAS.^11^ Note that *K*_aw_ for 21 PFAS in ref (5) was not considered here to avoid duplicate data use, as these data were derived from *K*_Hxd/w_ and *K*_Hxd/air_ through the thermodynamic cycle calculation.

Key to the accurate determination of the polar interaction descriptors is the availability of *K* data for partition systems with water as a partition phase because water is a strong H-bond donor and acceptor phase and thus the corresponding *K* values are sensitive to the H-bonding properties of the solute.^27^ In particular, at least one solvent/water or air/water partition coefficient is required to determine *B* because there is no commonly used GC phase that is a significant H-bond donor. In the data set, *K*_Hxd/w_ is sensitive to both *A* and *B* and *K*_ow_ is sensitive to *B*. However, *K*_Hxd/w_ and *K*_ow_ data are not available for all PFAS. To increase the number of PFAS for which descriptors can be determined, the following assumptions were made. First, PFAS that have the same nonfluorinated structure and differ only in the perfluoroalkyl chain length share the values of *S*, *A*, and *B*. Examples of this case are 4:2, 6:2, 8:2, 10:2, and 12:2 FTOHs. Second, *A* and/or *B* were set to 0 if the compound did not have a functional group that can form an H-bond in the respective manner. For example, *A* and *B* were set to 0 for perfluoroalkanes (PFAs), perfluoroalkyl iodides (PFAIs), and FTOs; *A* was set to 0 for X:2 fluorotelomer iodides (FTIs), acrylates (FTACs), and methacrylates (FTMACs); and *B* was set to 0 for 1H,8H-perfluorooctane (1,8-DHPFO). All of these assumptions are tabulated in Table S4.

Initially, the PP-LFER system parameters were calibrated by using only the data for nonfluorinated reference compounds and used to determine the solute descriptors for PFAS, as described above. However, this straightforward approach resulted in inconsistent descriptor values for some PFAS, as indicated by relatively large standard errors of descriptor values or unexpected negative *A* or *B* values. The reason for this is likely the problem of extrapolation; the system parameters calibrated with only nonfluorinated compounds are not applicable to PFAS.^9,18^ To circumvent this problem, the system parameters of eq 1 and the solute descriptors for PFAS were determined iteratively in this work. The complete scheme of the iterative calculation is shown in Figure S1. In this iterative calculation, relatively data-rich PFAS (i.e., FTOHs, PFASAs, *N*-alkylperfluoroalkanesulfonamides (FASAs), *N*-alkylperfluoroalkanesulfonamidoethanols (FASEs), PFAIs, FTIs, FTOs, FTACs, and FTMACs; see Table S5) were included in the calibration sets and used to tentatively calibrate the system parameters. The *S*, *A*, and *B* descriptor values predicted by the IFS-QSAR of EAS-E Suite^28^ were used as initial values for PFAS. The obtained system parameters were then used to update the PFAS descriptors, and using these updated PFAS descriptors, the system parameters were recalibrated, and so on. This cycle was repeated 20 times, which was sufficient to obtain stable results. *L* was also updated from the reported values^18^ in this iterative calculation using the literature data for nonpolar GC columns (squalane, SPB-Octyl,^18^ and Apolane)^11^ because small influences of polar interactions on log *k*, which were not considered in the previous studies,^11,18^ could be taken into account with the values of *S* and *A* for PFAS. The differences from the previous values, however, were small.

### Prediction by COSMO*therm*

The partition coefficients were predicted by the COSMO*therm* algorithm, which follows the COSMO-RS theory^29^ and can derive activity-related properties including partition coefficients, based on quantum chemical and statistical thermodynamic calculations. The programs Turbomole, COSMO*ConfX*, and COSMO*thermX* (version 2021, COSMO*logic*, Dassault Systèmes) were used for the calculation. More details about the method have been explained elsewhere.^5,18^

## Results and Discussion

### Isothermal GC Retention Behavior of Neutral PFAS

A total of 529 and 642 log *k* data were measured for PFAS and nonfluorinated reference compounds, respectively (Tables S6 and S7, Figure S2–S5). For each column and compound, log *k* was linear against 1/*T* (Figure S6), confirming the high measurement consistency over temperature. Log *k* data for a given class of PFAS (e.g., X:2 FTOHs) for the HP-5ms and DB-200 columns were perfectly linear against the number of CF_2_ units (Figure S7). However, the relationship for DB-225ms was very slightly concave upward, and it was more so for SolGel-WAX. Since linearity is a prerequisite for PP-LFER model fitting, the log *k* data for SolGel-WAX were not used for solute descriptor determination. For polar stationary phases, the contribution of interfacial adsorption to retention, in addition to the bulk partition mechanism, has been shown to be significant in some cases,^30^ which may be a reason for the observed nonlinearity.

**Figure 1:**
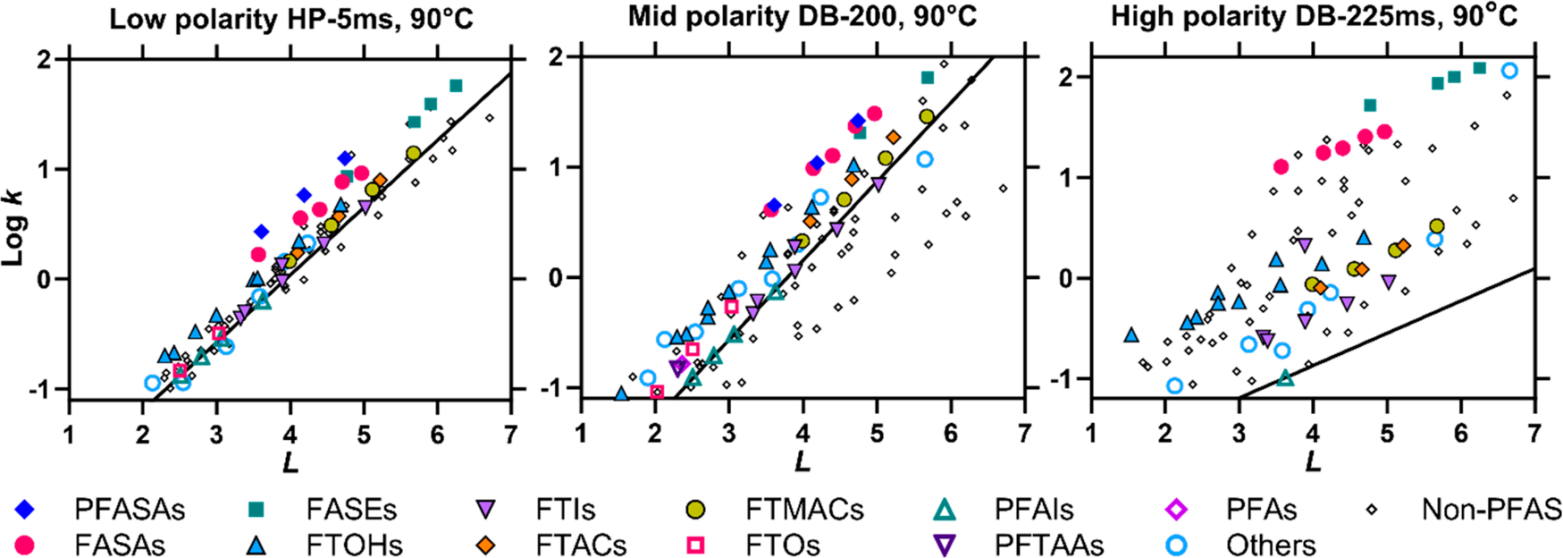
Examples of log GC retention factors (log *k*) against log hexadecane/air partition coefficients (*L*). The lines indicate the nonpolar PFAS baselines generated with the mean of the slopes for PFAIs, FTOs, and FTIs and the intercept adjusted to the data for PFAIs. Data for other temperatures are shown in Figures S2–S5.

The log *k* values for the low-polar HP-5ms column show a high correlation with *L* (Figure 1). Clearly, the nonpolar van der Waals interactions are prevalent with HP-5ms, and additional polar interactions have only minor influences on log *k*. The correlation between log *k* for the DB-200 column and *L* is less good, and a high scatter is evident for the highly polar DB-225ms and SolGel-Wax columns. For all columns, log *k* for nonpolar PFAS (i.e., without a polar functional group, e.g., PFAs, PFAIs, and FTOs) shows a linear relationship with *L* and forms a baseline in the log *k*–*L* plot. Other PFAS deviate upward from the PFAS baseline, depending on their polarities. This is most evident in the DB-225ms and SolGel-WAX data; PFASAs and their *N*-substituted compounds are farthest from the nonpolar PFAS in the plot, reflecting their strong polarity, followed by the moderately polar FTOHs. The other fluorotelomer compounds (i.e., FTACs, FTMACs, and FTIs) deviate slightly from the baseline, reflecting their weak polarity. The GC retention measurement is therefore useful to roughly assess the polarity of PFAS. The relevant types of polar interactions are discussed quantitatively in the following section.

### *K*_ow_ Measurement

The measured *K*_ow_ values are presented in Table 2 along with the literature data for three FTOHs. This data set covers most of the target PFAS classes that are expected to have both *A* and *B* > 0 and for which the availability of *K* data is particularly important for descriptor determination.

### Solute Descriptor Determination

The final adjusted solute descriptors for all PFAS are presented in Table S8, and those for selected PFAS are shown in Table 3. Table S8 also shows the number of data available for each PFAS or PFAS group. The system parameters for all GC columns and partition systems are given in Table S9. All five solute descriptors are now available for 47 neutral PFAS. For the remaining 13 PFAS, the values of *S*, *A*, *V*, and *L* are available, but the value of *B* is missing due to a lack of *K* data. The quality of solute descriptor fitting was high, with SDs generally <0.1 log units. The relatively high SDs (0.1–0.5) were obtained only for PFAs, FEs, and perfluorotrialkylamines (PFTAAs), suggesting that the uncertainty of the descriptor values for these PFAS may be high. The reason for the low fit is currently unknown but could be related to the particularly high degree of fluorination of these PFAS, with little or no nonfluorinated substructure in the molecules.

**Table 3:** Solute Descriptor Values (±SE) for Selected PFAS Determined in This Study

	*S*	*A*	*B*
X:1 FTOHs	0.30 ± 0.02	0.84 ± 0.02	0.14 ± 0.01
X:2 FTOHs	0.35 ± 0.02	0.60 ± 0.01	0.31 ± 0.01
5:2s FTOH	0.39 ± 0.02	0.62 ± 0.02	0.19 ± 0.01
PFASAs	0.96 ± 0.03	1.15 ± 0.03	0.23 ± 0.02
*N*-alkyl-FASAs	0.88 ± 0.02	0.74 ± 0.02	0.24 ± 0.01
*N*-alkyl-FASEs	1.00 ± 0.02	0.55 ± 0.03	0.68 ± 0.02
PFAIs	0.07 ± 0.01	0	0
X:2 FTIs	0.32 ± 0.01	0	0.17 ± 0.01
6:1 FTI	0.27 ± 0.02	0.13 ± 0.02	0.11 ± 0.01
6:1 FTI-7H	0.63 ± 0.04	0.24 ± 0.04	0.14 ± 0.02
PFAs	–0.19 ± 0.06	0	0
X:2 FTOs	0.11 ± 0.01	0	0
X:2 FTACs	0.54 ± 0.01	0	0.40 ± 0.01
X:2 FTMACs	0.53 ± 0.01	0	0.39 ± 0.01

### Solute Descriptor Values and Molecular Structure

**Figure 2:**
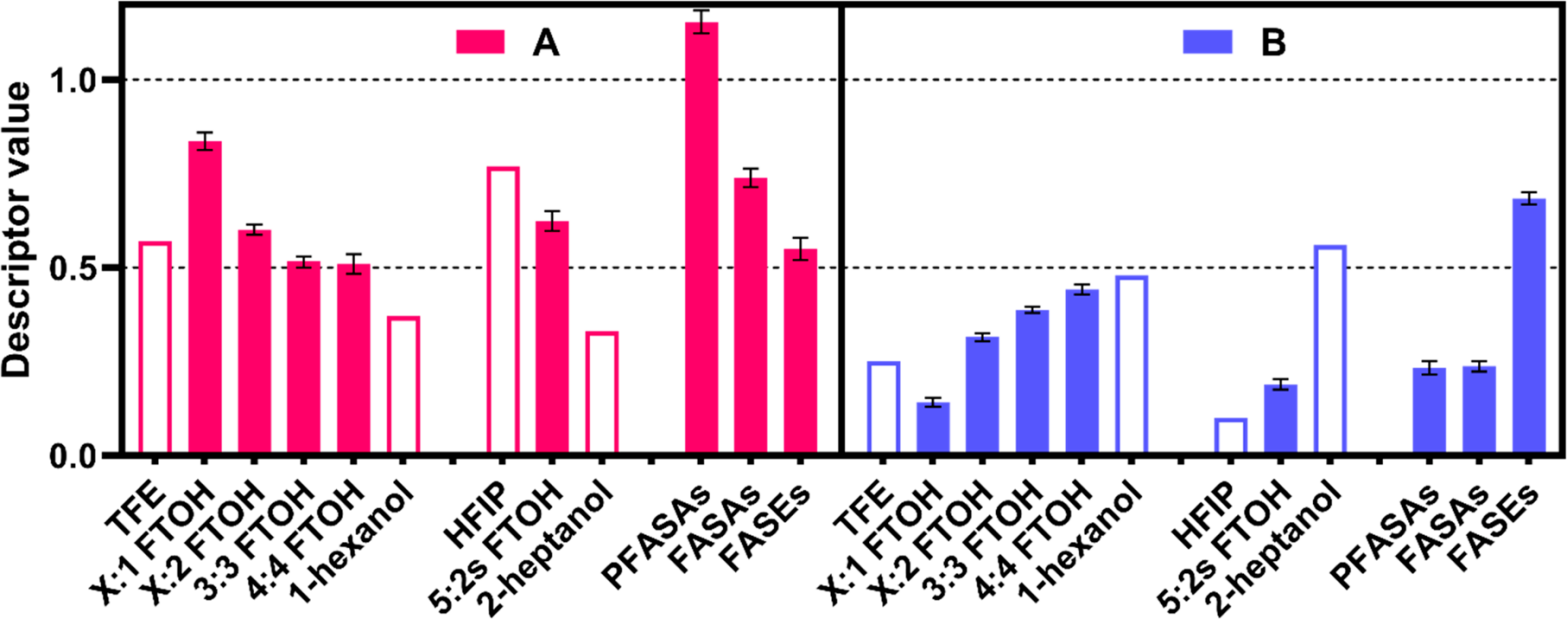
Solute H-bond donor (*A*) and acceptor (*B*) properties of selected PFAS. Solid bars are data from this study, and blank bars are data from the literature. Error bars indicate standard errors. 2,2,2-trifluoroethanol (TFE), 1,1,1,3,3,3-hexafluoro-2-propanol (HFIP).

The *A* and *B* descriptors for FTOHs are higher and lower, respectively, than those for nonfluorinated alkan-1-ols (Figure 2), consistent with previous reports for X:2 FTOHs.^9,11,12^ The secondary alcohol 5:2s FTOH also has high *A* and low *B* values. The increased H-bond acidity and decreased H-bond basicity of fluorinated alcohols such as 2,2,2-trifluoroethanol and 1,1,1,3,3,3-hexafluoropropan-2-ol have long been known^31,32^ and can be explained by the electron-withdrawing effect of F on the −OH group. The results of this study demonstrate that this effect of F depends on the −CH_2_– spacer length of FTOHs. Thus, 3:1 and 7:1 FTOHs (only one −CH_2_– spacer) have the highest *A* and lowest *B* values of the FTOHs studied. As the spacer length increases, the influence of F on −OH diminishes and the *A* and *B* values approach those for nonfluorinated alcohols.

Not only FTOHs, but other PFAS also tend to have higher *A* values than their nonfluorinated counterparts, and the opposite is true for *B* values. For example, the *B* values of alkyl acrylates and methacrylates in the ABSOLV database are 0.42–0.55 and those of 1-iodoalkanes are 0.14–0.17.^10^ These values are ∼0.1 lower than those of the corresponding fluorotelomer compounds. There are no descriptors for nonfluorinated alkanesulfonamides, but the descriptors for nonfluorinated benzenesulfonamides are available in the database. None of the variously substituted benzenesulfonamides have an *A* value greater than 1 or a *B* value less than 0.7, suggesting that the *A* (1.15) and *B* (0.26) values for PFASAs obtained in this work are significantly influenced by the perfluoroalkyl structure.

The descriptors of PFASAs, FASAs, and FASEs indicate that the *A* value decreases with the number of substitutions on the N atom (i.e., −SO_2_NH_2_ > −SO_2_NHR > −SO_2_N(C_2_H_5_OH)R). The significant H-bond donor property of FASEs should be due to their hydroxyethyl group since the N atom does not have an H atom. The *B* values for PFASAs and FASAs are relatively low, and that of FASEs is higher due to the hydroxyethyl group.

In a previous article,^5^ it was shown that 1H,1H,7H-perfluoroheptyl iodide (6:1 FTI-7H) has a relatively high affinity for water compared to 6:1 FTI. The electron-withdrawing F atoms were thought to make the H atom at the terminal methyl group of 6:1 FTI-7H significantly H-bond donating.^5^ In fact, the *A* of 6:1 FTI-7H is positive (0.24) and higher than that of 6:1 FTI (0.13). However, incomplete fluorination affects not only *A* but also the *S* and *L* values. The *S* and *L* values of 6:1 FTI-7H are 0.63 and 3.89, repectively, and are higher than those of 6:1 FTI (0.27 and 3.38, respectively), although the size (*V*) of the two compounds is similar. A term-by-term analysis of the PP-LFER model indicates that the higher *S* of 6:1 FTI-7H than 6:1 FTI contributes more to the lower *K*_aw_ and *K*_Hxd/w_ values of 6:1 FTI-7H. Similarly, 1,8-DHPFO has *A*, *S*, and *L* values that are 0.24, 0.27, and 0.78 higher, respectively, than its perfluorinated counterpart, perfluorooctane. Increased *S* and *L* mean that partially hydrogenated PFAS interact more strongly with any phase than the fully fluorinated analogs do. Thus, even one or two H substitutions on the perfluoroalkyl chain make the molecule significantly more interactive, reducing the characteristically weak interaction properties of PFAS.

### Comparison to COSMO*therm* Predictions

**Figure 3:**
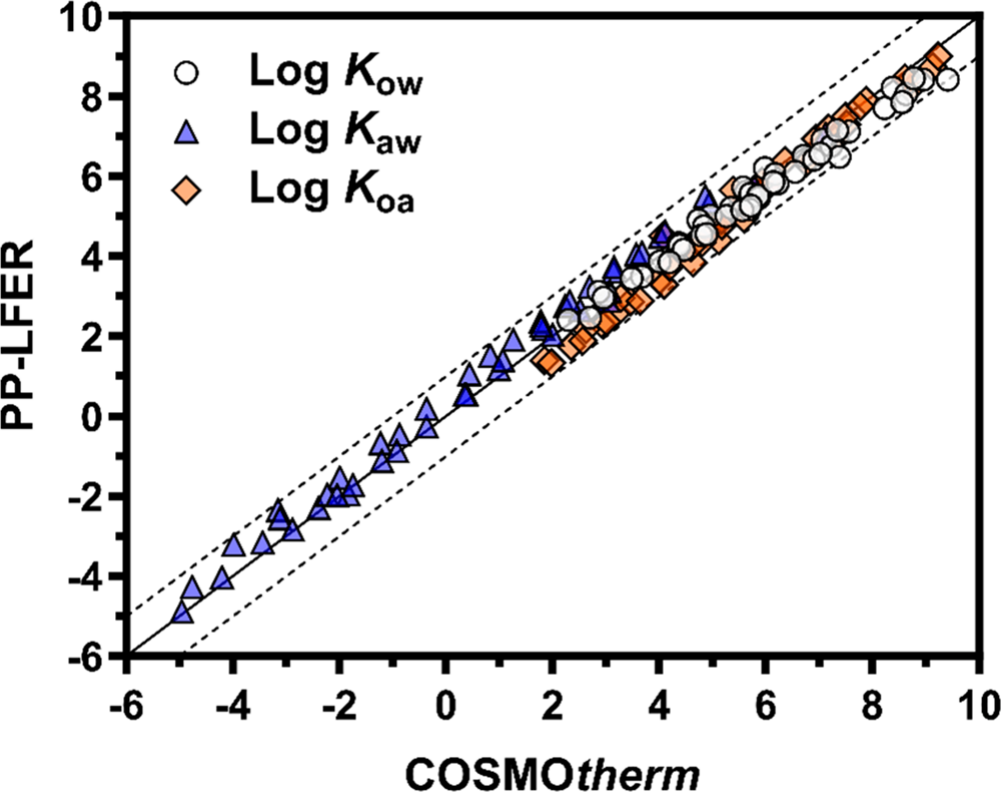
Partition coefficients predicted by PP-LFERs and COSMO*therm*. The solid lines indicate the 1:1 agreement, and the dashed lines indicate the ±1 log unit deviation.

Using the PP-LFER solute descriptors for PFAS and the system parameters obtained above, we calculated values for *K*_ow_, *K*_aw_, and *K*_oa_ for the 47 neutral PFAS. Ideally, these predictions should be compared to external experimental data to validate the prediction accuracy. However, such data are not available. As an alternative approach, the predictions from the PP-LFERs were compared to those from COSMO*therm* (Figure 3). The root mean squared errors (RMSE) for log *K*_ow_, log *K*_aw_, and log *K*_oa_ were 0.37, 0.42, and 0.33, respectively, indicating high agreement. Since the two prediction models are based on completely different principles, the agreement increases the reliability of both models. COSMO*therm* provided, on average, 0.26 (±0.27) and 0.24 (±0.23) higher log *K*_ow_ and log *K*_oa_ predictions, respectively, and 0.33 (±0.25) lower log *K*_aw_ values than the PP-LFERs. It is likely that the current version of COSMO*therm* slightly overestimates the partitioning of PFAS from air to liquid phases, as reported in previous articles.^5,18^ Note that some predictions from the PP-LFERs presented here cannot be considered “fully predicted” because the solute descriptors used were calibrated with the experimental data for *K*_ow_ and *K*_aw_. Further comparison between PP-LFERs and COSMO*therm* was performed for other solvent/water and solvent/air partition coefficients using the PP-LFER equations published in the literature^8,33^ (Figure S8). Although the PP-LFER equations in the literature are often not calibrated with PFAS, the predictions of PP-LFERs and COSMO*therm* agree within 1 log unit in most cases, similar to the results shown in Figure 3.

For comparison, the predictions from the EPI-Suite software provided by the U.S. EPA^34^ were plotted against those from COSMO*therm* (Figure S9). The RMSE values for log *K*_ow_, log *K*_aw_, and log *K*_oa_ were 0.27, 2.50, and 2.56, respectively, showing a large discrepancy for *K*_aw_ and *K*_oa_. This result suggests that *K*_aw_ and *K*_oa_ predictions by EPI-Suite are inaccurate for many neutral PFAS.

### Chemical Partitioning Space Plot

**Figure 4:**
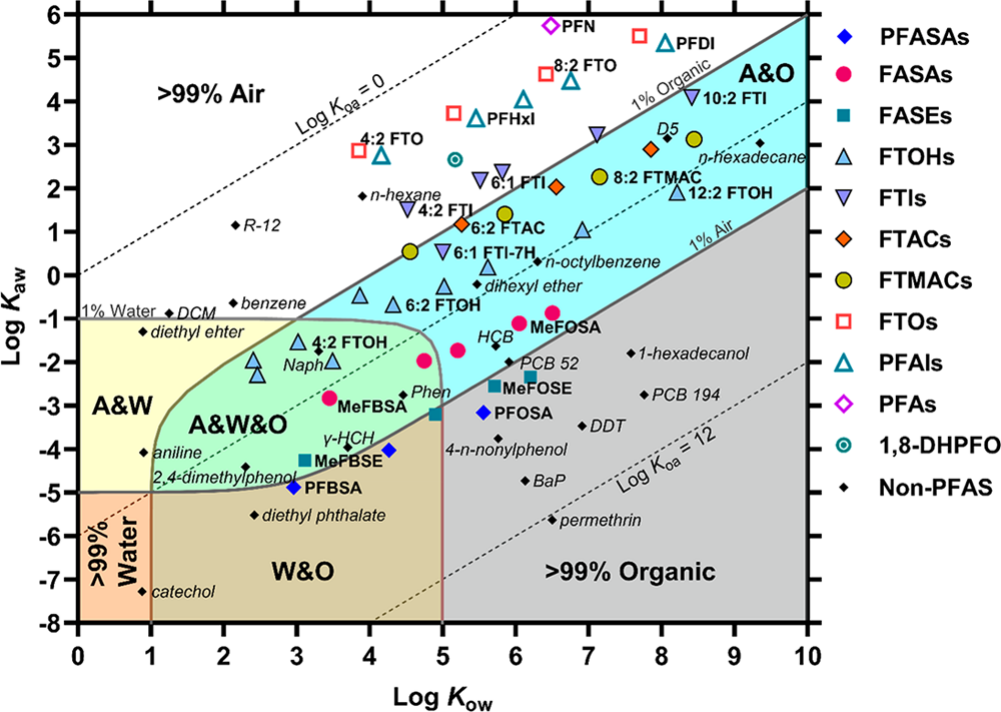
Chemical partitioning space plot for neutral PFAS. The volumes of air, water, and organic phase (represented by octanol) were 10^6^:10^3^:1. Solid lines indicate 1% by mass of the chemicals in the respective phases. Dashed lines indicate log *K*_oa_ values of 0, 6, and 12. Partition coefficients for PFAS were calculated by the PP-LFERs from this study, and those for the non-PFAS reference compounds were experimental values from the literature^22,34,40−43^ or predicted by the PP-LFERs.^10^ The acronyms indicate as follows: dichlorodifluoromethane (R-12), dichloromethane (DCM), decamethylcyclopentasiloxane (D5), γ-hexachlorocyclohexane (γ-HCH), naphthalene (Naph), phenanthrene (Phen), benzo[*a*]pyrene (BaP), hexachlorobenzene (HCB), polychlorinated biphenyl (PCB), and dichlorodiphenyltrichloroethane (DDT). Perfluorododecane is out of scale (log *K*_ow_ = 8.4, log *K*_aw_ 7.1).

To provide an overview of the environmental phase partition properties of neutral PFAS, a chemical partitioning space plot was generated following the approach in the literature^35,36^ (Figure 4). The figure plots log *K*_aw_ against log *K*_ow_, both predicted by the PP-LFERs, and shows the equilibrium mass fractions of PFAS in a hypothetical system consisting of air, water, and an organic solid (represented by octanol). The volume ratios of these phases were assumed to be 10^6^:10^3^:1, respectively, representing the environment as a whole.^35,36^ Note that interfacial adsorption was not considered here for simplicity and the lack of parameters.

The 47 neutral PFAS are broadly distributed in the plot, reflecting the diversity of the properties. A general trend is that neutral PFAS prefer the air phase; the equilibrium mass in air is >99% for 16 and at least 1% for 41 of the 47 compounds. In addition, water is often an unpreferred phase; the equilibrium mass in water is >1% for only 10 PFAS. These features are well expected due to the weak van der Waals interactions with any phase and the high cavity energy in water of PFAS.

Looking at specific classes of PFAS, the nonpolar PFAS are all >99.9% in the air phase, reflecting their extremely high volatility. Non-PFAS contaminants with similar phase distribution properties may be limited to alkanes and refrigerant compounds (e.g., R-12). Weakly polar PFAS (e.g., FTIs, FTACs) are primarily found in air, but with an appreciable organic phase contribution (0.1–20%). The more polar FTOHs are more abundant in the organic phase (2–70%). These distribution patterns of fluorotelomer compounds are comparable to those of medium to long alkyl compounds with no or a weakly polar functional group (e.g., *n*-hexadecane, dihexyl ether) and, notably, cyclic volatile methylsiloxanes (e.g., D5). PFASAs and their derivatives are even more in the organic phase, similar to moderately hydrophobic persistent organic pollutants (POPs), e.g., hexachlorocyclohexanes, hexachlorobenzene, and polychlorinated biphenyls with low degree of chlorination. Only short-chain PFAS with a polar functional group may be present in water at >1%. Note that PFASAs and FASAs are weak acids and may ionize in water and partition more into water,^5^ which is not accounted for in the plot. Figure S10 shows a plot for 134 neutral PFAS using the log *K*_aw_ and log *K*_ow_ values predicted by COSMO*therm*. These neutral PFAS are from the lists of Kissel et al.^37^ and our previous study.^18^ The data points are even more widely distributed, although the plot again shows that air is the most important phase and that the air/organic phase distribution can be an important fate determinant, except for short-chain polar PFAS, which can also be in water.

An important question is the extent to which octanol can represent environmental organic phases for neutral PFAS. Available data for FTOHs suggest that soil organic carbon/water partition coefficients are ∼2 log units lower than *K*_ow_.^38^ If this is generally the case for neutral PFAS, then the organic phase contribution in Figure 4 is overestimated.

This study determined the PP-LFER solute descriptors of neutral PFAS that can be used to predict various partition coefficients. As noted above, many PP-LFER equations in the literature have not been calibrated with PFAS. Extrapolation may reduce prediction accuracy from what could otherwise be achieved.^39^ This is not a problem specific to the PP-LFER models. Any empirical model requires high-quality data on PFAS to accurately predict PFAS properties, but such data are often lacking. In our series of studies, we measured *K*_ow_, *K*_Hxd/w_, and *K*_Hxd/air_ for various neutral PFAS, which can also provide *K*_aw_ and *K*_oa_ values via thermodynamic cycles. *K*_oa_, *K*_oil/w_, organic carbon/water, and liposome/water partition coefficients can be found in the literature for some FTOHs.^9^ Measuring additional partition data for other PFAS or other partition systems would expand the applicability of the PP-LFER approach for PFAS. COSMO*therm* is a theoretical model and may have the potential to provide accurate predictions without an additional empirical calibration. However, COSMO*therm* is not free of charge and sometimes requires a long computation time for quantum chemical calculations, in contrast to the PP-LFER model. In the Supporting Information, COSMO*therm* predictions for *K*_ow_, *K*_aw_, *K*_oa_, *K*_Hxd/w_, *K*_Hxd/air_ (*L*), *K*_Hxd/w_, *K*_oil/w_, and *K*_oil/air_ of 222 neutral (or neutral species of acidic) PFAS are provided for reference (Table S10). While reasonable accuracy (within ∼1 log unit) is expected, as discussed above, further validation of these predictions with experimental data is warranted.

## Supporting Information Available

Description of the measurement of *K*_ow_; tables listing the PFAS and reference compounds used, solute descriptors, measured log *k* values for PFAS, PP-LFER system parameters, and log *K* values predicted by COSMO*therm*; and figures for schematic illustration of the iterative calculation procedure of descriptors and parameters, measured log *k* vs *L*, vs the number of CF_2_ units, and vs the reciprocal of temperature, predictions by PP-LFERs vs COSMO*therm* for 47 PFAS, predictions by EPI-Suite and COSMO*therm*, and a chemical partitioning space plot for 134 neutral PFAS (PDF)
