# Supplementary material for: Intermolecular Interactions, Solute Descriptors, and Partition Properties of Neutral Per- and Polyfluoroalkyl Substances (PFAS)
Source: Environ Sci Technol. 2023 Nov 1;57(45):17534–41. doi: 10.1021/acs.est.3c07503 (PMC10653080; doi:10.1021/acs.est.3c07503)
Supplement: Supplementary file 1 — es3c07503_si_001.pdf [file es3c07503_si_001.pdf]

Supporting Information for "Intermolecular Interactions, Solute Descriptors, and Partition Properties of Neutral Per- and Polyfluoroalkyl Substances (PFAS)"

Satoshi Endo

Health and Environmental Risk Division, National Institute for Environmental Studies (NIES), Onogawa 16-2, 305-8506 Tsukuba, Ibaraki, Japan

Tel: +81-29-850-2695, email: endo.satoshi@nies.go.jp

Summary: 38 pages, 10 figures, and 10 tables.

Content

SI-1 Measurement of octanol/water partition coefficient (K_{ow})

Table S1. List of PFAS used in this study.

Table S2. List of reference compounds.

Table S3. Solute descriptors for reference compounds.

Table S4. Fixed and adjusted values of solute descriptors.

Table S5. Selected PFAS included in the calibration sets for PP-LFER models.

Table S6. The measured log *k* values for PFAS.

Table S7. The measured log *k* values for reference compounds.

Table S8. Finally adjusted solute descriptors for PFAS.

Table S9. PP-LFER system parameters obtained in this study.

Table S10. Log *K* values of neutral (species of) PFAS predicted by COSMO*therm* (25°C, unless otherwise noted).

Figure 1. Schematic illustration of the iterative calculation procedure of PFAS solute descriptors and system parameters.

Figure S2. Measured log *k* with HP-5ms vs log $K_{Hxd/air}$ (*L*).

Figure S3. Measured log *k* with DB-200 vs log $K_{Hxd/air}$ (*L*).

Figure S4. Measured log *k* with DB-225ms vs log $K_{Hxd/air}$ (*L*).

Figure S5. Measured log *k* with SolGel-WAX vs log $K_{Hxd/air}$ (*L*).

Figure S6. Log *k* against the reciprocal of temperature for selected PFAS.

Figure S7. Log *k* against the number of CF_2 units for selected groups of PFAS.

Figure S8. Predictions by PP-LFERs vs COSMO*therm* for 47 PFAS.

Figure S9. Partition coefficients predicted by EPI-Suite and COSMO*therm*.

Figure S10. Chemical partitioning space plot for 134 neutral PFAS using COSMO*therm*-predicted partition coefficients.

SI-1 Measurement of octanol/water partition coefficient (K_{ow})

K_{ow} was measured for nine PFAS using shared-headspace and batch partition methods,[1] as described below.

Shared-headspace method

K_{ow} values for six relatively volatile PFAS, namely 3:3, 4:4, 7:1, and 5:2s FTOHs, as well as 6:1 FTI and 6:1 FTI-7H, were determined using a shared-headspace method. A water-saturated octanol solution of the compound (300 μL) was put in a 350-μL glass insert, which was inserted in a 1.5-mL vial and placed together in a 10-mL headspace vial containing 1 mL of octanol-saturated water. Five replicate vials were prepared. The concentration in the octanol solution was 100–4000 mg/L, depending on the compounds. A pair of compounds (3:3 and 4:4 FTOHs, 7:1 and 5:2s FTOHs, and 6:1 FTI and 6:1 FTI-7H) were mixed in octanol solution and measured simultaneously. The 10-mL vial was sealed with a PTFE-lined septum and shaken at 25 °C for 24 h. The water in the 10-mL vial was then collected and immediately extracted with *n*-hexane. Prior to gas chromatography/mass spectrometry (GC/MS) analysis, an internal standard (6:2 FTOH or 6:2 FTI) was added to the *n*-hexane extracts. K_{ow} was obtained from the measured concentration in water and the concentration in octanol, the latter was assumed to be unchanged from the prepared concentration due to limited partitioning of the studied compounds from octanol to air and water.

Batch partition method

A batch partition method was adapted to measure K_{ow} of PFBSA, MeFBSA, and MeFBSE. A 10-mL glass screw vial received 5 mL of 1 mM HCl water (for PFBSA and MeFBSA) or pure water (for MeFBSE) and 5 mL of an octanol solution of individual PFAS (10 or 100 mg/L). Five replicate vials were prepared. The vials were closed with PTFE-lined septa and shaken for 24 h (60 rpm, 25 °C). The vials were then collected, flipped upside down and held still for 24 h. The water in the vial was then sampled through the septum with a syringe and subjected to liquid chromatography/mass spectrometry (LC/MS) analysis. K_{ow} was calculated from the measured water phase concentration, as described above.

Instrumental analysis

The methods for GC/MS and LC/MS analyses were described in detail in the previous publication.[1] Briefly, a 7890A/5975C GC/MS system (Agilent Technologies) equipped with an MPS2 autosampler and a CIS4 injector (Gerstel) was used. The analytical column used was Rtx-624 (0.25 mm × 60 m, 1.4 μm film thickness, Restek). Helium was used as the carrier gas (1.2 mL/min). The oven temperature was increased from 50 to 230 °C in a varying temperature program. The sample (1 or 2 μL) was injected into the GC in cold splitless injection mode. The MS detector was operated in the selected ion monitoring (SIM) mode. Negative electrospray ionization (ESI) LC/MS analysis was performed using a 1260 Infinity II LC with a single quadrupole MS detector (Agilent Technologies). A Kinetex EVO

C18 column (2.6 µm particle size, 50 × 2.1 mm i.d., Phenomenex) was used for separation. The sample injection volume was 2 µL, and a gradient elution was adapted with (A) methanol and (B) water containing 10 mM ammonium formate. MS conditions were as follows: Gas temperature, 350 °C; drying gas flow, 12 L/min; nebulizer pressure, 40 psig; quadrupole temperature, 100 °C; capillary voltage, 3000 V; fragmentor voltage, 50 V. Samples were measured in SIM mode for quantification.

Table S1. List of PFAS used in this study.

Name	Abbreviation	CAS-RN	Group	Provider	Purity (%)
1H,1H-Perfluorobutan-1-ol	3:1 FTOH	375-01-9	FTOHs	TCI	98
4,4,5,5,6,6,6-Heptafluorohexan-1-ol	3:3 FTOH	679-02-7	FTOHs	Apollo Scientific	95
1H,1H,2H,2H-Perfluorohexan-1-ol	4:2 FTOH	2043-47-2	FTOHs	TCI	97
5,5,6,6,7,7,8,8,8-Nonafluorooctan-1-ol	4:4 FTOH	3792-02-7	FTOHs	SynQuest	?
1H,1H,2H,2H-Perfluorooctan-1-ol	6:2 FTOH	647-42-7	FTOHs	TCI	98
1H,1H-Perfluorooctan-1-ol	7:1 FTOH	307-30-2	FTOHs	SynQuest	98
1H,1H,2H,2H-Perfluorodecan-1-ol	8:2 FTOH	678-39-7	FTOHs	TCI	97
1H,1H,2H,2H-Perfluorododecan-1-ol	10:2 FTOH	865-86-1	FTOHs	SynQuest	97
1H,1H,2H,2H-Perfluorotetradecan-1-ol	12:2 FTOH	39239-77-5	FTOHs	SynQuest	?
1H,1H,1H,2H-Perfluoroheptan-2-ol	5:2s FTOH	914637-05-1	FTOHs	SynQuest	97
N-Ethylperfluorohexane-sulfonamidoethanol	EtFHxSE	34455-03-3	FASEs	SynQuest	?
N-Ethylperfluorooctane-sulfonamidoethanol	EtFOSE	1691-99-2	FASEs	SynQuest	95(sum of isomers)
N-Methylperfluorobutane-sulfonamidoethanol	MeFBSE	34454-97-2	FASEs	TRC	?
N-Methylperfluorooctane-sulfonamidoethanol	MeFOSE	24448-09-7	FASEs	SynQuest	95(sum of isomers)
Perfluorobutanesulfonamide	PFBSA	30334-69-1	FASAs	SynQuest	97
Perfluorohexanesulfonamide	PFHxSA	41997-13-1	FASAs	SynQuest	95(sum of isomers)
Perfluorooctanesulfonamide	PFOSA	754-91-6	FASAs	SynQuest	85(sum of isomers)
N-Methylperfluorobutanesulfonamide	MeFBSA	68298-12-4	FASAs	SynQuest	97
N-Methylperfluorohexanesulfonamide	MeFHxSA	68259-15-4	FASAs	SynQuest	97
N-Methylperfluorooctanesulfonamide	MeFOSA	31506-32-8	FASAs	TRC	?
N-Ethylperfluorohexanesulfonamide	EtFHxSA	87988-56-5	FASAs	SynQuest	97
N-Ethylperfluorooctanesulfonamide	EtFOSA	4151-50-2	FASAs	SynQuest	95
Perfluorobutanesulfonyl fluoride	PFBSF	375-72-4	Others	SynQuest	97
Perfluorobutyl iodide	PFBI	423-39-2	PFIs	TCI	98
Perfluorohexyl iodide	PFHxI	355-43-1	PFIs	SynQuest	98
Perfluoroheptyl iodide	PFHpI	335-58-0	PFIs	SynQuest	97
Perfluorooctyl iodide	PFOI	507-63-1	PFIs	SynQuest	98
Perfluorodecyl iodide	PFDI	423-62-1	PFIs	TCI	98
1H,1H,2H,2H-Perfluorohexyl iodide	4:2 FTI	2043-55-2	FTIs	TCI	99
1H,1H-Perfluoroheptyl iodide	6:1 FTI	212563-43-4	FTIs	Fujifilm-Wako	97
1H,1H,7H-Perfluoroheptyl iodide	6:1 FTI-7H	376-32-9	FTIs	Fluorochem	98
1H,1H,2H,2H-Perfluorooctyl iodide	6:2 FTI	2043-57-4	FTIs	TCI	97
1H,1H,2H,2H-Perfluorodecyl iodide	8:2 FTI	2043-53-0	FTIs	TCI	98

1H,1H,2H,2H-Perfluorododecyl iodide	10:2 FTI	2043-54-1	FTIs	Sigma-Aldrich	95
1,1,1,2,2,3,3-Heptafluoro-3-[(1,1,1,2,3,3-hexafluoro-3-{[1,1,1,2,3,3-hexafluoro-3-(1,2,2,2-tetrafluoroethoxy)-2-propanyl]oxy}-2-propanyl)oxy]propane	FE-E3	3330-16-3	FEs	SynQuest	95(99)
1,1,1,2,4,4,5,7,7,8,10,10,11,13,13,14,14,15,15,15-Eicosafluoro-5,8,11-tris(trifluoromethyl)-3,6,9,12-tetraoxapentadecane	FE-E4	26738-51-2	FEs	SynQuest	97
1,1,1,2,4,4,5,7,7,8,10,10,11,13,13,14,16,16,17,17,18,18,18-Tricosafluoro-5,8,11,14-tetrakis(trifluoromethyl)-3,6,9,12,15-pentaoxaoctadecane	FE-E5	37486-69-4	FEs	TCI	95
Allyl 1H,1H-perfluorooctyl ether	AFOE	812-72-6	Others	SynQuest	97
1-(Heptafluoropropoxy)-1,2,2,2-tetrafluoro-1-iodoethane	FE-E1-I	107432-46-2	Others	Apollo Scientific	97
Allyl perfluoroisopropyl ether	APFIPE	15242-17-8	Others	SynQuest	95
Perfluorotripropyl amine	PFTPrA	338-83-0	PFTAAs	Combi-Blocks	95
Perfluorotributyl amine	PFTBA	311-89-7	PFTAAs	Fujifilm-Wako	65 (MS calibrant)
Perfluorononane	PFN	375-96-2	PFAs	Fujifilm-Wako	98
Perfluorododecane	PFDoD	307-59-5	PFAs	SynQuest	97
1H,8H-Perfluorooctane	1,8-DHPFO	307-99-3	Others	SynQuest	97
1,8-Divinylperfluorooctane	1,8-DVPFO	35192-44-0	Others	SynQuest	95
4-(Perfluorooct-1-yl)styrene	PFOSt	106209-21-6	Others	SynQuest	97
1H,1H,2H-Perfluoro-1-hexene	4:2 FTO	19430-93-4	FTOs	TCI	98
1H,1H,2H-Perfluoro-1-octene	6:2 FTO	25291-17-2	FTOs	TCI	96
1H,1H,2H-Perfluoro-1-decene	8:2 FTO	21652-58-4	FTOs	TCI	97
1H,1H,2H-Perfluoro-1-dodecene	10:2 FTO	30389-25-4	FTOs	Alfa Aesar	97
1H,1H,2H,2H-Perfluorooctyl acrylate	6:2 FTAC	17527-29-6	FTACs	TCI	98
1H,1H,2H,2H-Perfluorodecyl acrylate	8:2 FTAC	27905-45-9	FTACs	TCI	97
1H,1H,2H,2H-Perfluorododecyl acrylate	10:2 FTAC	17741-60-5	FTACs	Sigma-Aldrich	96
1H,1H,2H,2H-Perfluorohexyl methacrylate	4:2 FTMAC	1799-84-4	FTMACs	TCI	98
1H,1H,2H,2H-Perfluorooctyl methacrylate	6:2 FTMAC	2144-53-8	FTMACs	TCI	98
1H,1H,2H,2H-Perfluorodecyl methacrylate	8:2 FTMAC	1996-88-9	FTMACs	TCI	98
1H,1H,2H,2H-Perfluorododecyl methacrylate	10:2 FTMAC	2144-54-9	FTMACs	TRC	?
4-(1H,1H,2H,2H-Perfluorooctyl)benzyl alcohol	6:2 FTBnOH	356055-76-0	Others	Sigma-Aldrich	97
1H,1H,2H,2H-Perfluorodecyl acetate	8:2 FTAce	37858-04-1	Others	Wellington	dilution

Table S2. List of reference compounds.

Name	CAS-RN	Provider	Purity (%)
2-Methylpropane	75-28-5	STYLE	In Prince GB-2001 LPG
n-Butane	106-97-8	STYLE	In Prince GB-2001 LPG
Acetone	67-64-1	Fujifilm-Wako	99.5
Acetonitrile	75-05-8	Fujifilm-Wako	99.8
Propan-2-ol	67-63-0	Fujifilm-Wako	99.7
2-Methylbutane	78-78-4	STYLE	In Prince GB-2001 LPG
Diethyl ether	60-29-7	Fujifilm-Wako	99.5
Dichloromethane	75-09-2	Fujifilm-Wako	99.5
Propan-1-ol	71-23-8	Nacalai tesque	99.5
n-Pentane	109-66-0	STYLE	In Prince GB-2001 LPG
Butan-2-one	78-93-3	Fujifilm-Wako	99
Ethyl acetate	141-78-6	Fujifilm-Wako	99.7
Carbon disulfide	75-15-0	Fujifilm-Wako	99
Trichloromethane	67-66-3	Fujifilm-Wako	99
1,2-Dichloroethane	107-06-2	Fujifilm-Wako	99.5
Butan-1-ol	71-36-3	Nacalai tesque	99
Tetrahydrofuran	109-99-9	Fujifilm-Wako	99.5
n-Hexane	110-54-3	Fujifilm-Wako	96
Benzene	71-43-2	Fujifilm-Wako	99.7
1-Nitropropane	108-03-2	Fujifilm-Wako	97.0
Cyclohexane	110-82-7	Fujifilm-Wako	99.7
Pyridine	110-86-1	Fujifilm-Wako	99.5
Pentan-1-ol	71-41-0	Nacalai tesque	98
n-Pentylamine	110-58-7	TCI	98
Dimethylformamide	68-12-2	TCI	99.5
n-Heptane	142-82-5	Fujifilm-Wako	99.0
Toluene	108-88-3	Fujifilm-Wako	99.7
n-Butyl acetate	123-86-4	Fujifilm-Wako	99.0
Dimethyl sulfoxide	67-68-5	Fujifilm-Wako	?
Cyclohexanol	108-93-0	Sigma-Aldrich	99
Cyclohexanone	108-94-1	TCI	99
1,4-Butanediol	110-63-4	Fujifilm-Wako	98
Trimethyl phosphate	512-56-1	Fujifilm-Wako	99.0
Methyl phenyl ether	100-66-3	Fujifilm-Wako	99.0
Di-n-butyl ether	142-96-1	Fujifilm-Wako	98.0
Octamethyltrisiloxane (L3)	107-51-7	Sigma-Aldrich	98
2-Chlorophenol	95-57-8	Fujifilm-Wako	98.0
trans-Cyclohexane-1,2-diol	1460-57-7	Fujifilm-Wako	97
n-Nonane	111-84-2	Nacalai tesque	98.0
Octan-2-one	111-13-7	Fujifilm-Wako	98.0
Phenyl acetate	122-79-2	TCI	98
1-Nitrohexane	646-14-0	Sigma-Aldrich	98
Octamethylcyclotetrasiloxane (D4)	556-67-2	Fujifilm-Wako	95
1,2-Dichlorobenzene	95-50-1	TCI	99.0

Octan-1-ol	111-87-5	TCI	99.5
2,6-Dimethylphenol	576-26-1	TCI	99
n-Decane	124-18-5	Nacalai tesque	99.5
2-Phenylethanol	60-12-8	Fujifilm-Wako	98.0
Triethyl phosphate	78-40-0	Fujifilm-Wako	97.0
4-Chlorophenol	106-48-9	Fujifilm-Wako	98.0
Resorcinol	108-46-3	Fujifilm-Wako	99
1-Phenyl-1-propanol	93-54-9	TCI	98
n-Undecane	1120-21-4	Sigma-Aldrich	Alkane standard solution C8-C20
Decamethylcyclo pentasiloxane (D5)	541-02-6	TCI	99
1,2,4-Trichlorobenzene	120-82-1	Sigma-Aldrich	99
Benzamide	55-21-0	TCI	99.0
Decan-1-ol	112-30-1	TCI	98
4-Nitroanisole	100-17-4	TCI	98
n-Dodecane	112-40-3	Sigma-Aldrich	Alkane standard solution C8-C20
Methyl cinnamate	103-26-4	Fujifilm-Wako	98.0
1,3-Dinitrobenzene	99-65-0	Sigma-Aldrich	97
Dihexyl ether	112-58-3	Sigma-Aldrich	97
Dodecamethylcyclohexasiloxane (D6)	540-97-6	TCI	97
Tri-n-propyl phosphate	513-08-6	Sigma-Aldrich	99
Dodecan-2-one	6175-49-1	TCI	98
n-Tridecane	629-50-5	Sigma-Aldrich	Alkane standard solution C8-C20
Dimethyl phthalate	131-11-3	Fujifilm-Wako	98
4-Nitroaniline	100-01-6	Fujifilm-Wako	99
Dodecan-1-ol	112-53-8	TCI	99
n-Tetradecane	629-59-4	Sigma-Aldrich	Alkane standard solution C8-C20
Tetradecan-2-one	2345-27-9	TCI	97
n-Pentadecane	629-62-9	Sigma-Aldrich	Alkane standard solution C8-C20
n-Hexadecane	544-76-3	Sigma-Aldrich	Alkane standard solution C8-C20
n-Heptadecane	629-78-7	Sigma-Aldrich	Alkane standard solution C8-C20
n-Octadecane	593-45-3	Sigma-Aldrich	Alkane standard solution C8-C20
n-Dodecylbenzene	123-01-3	Sigma-Aldrich	Alkane standard solution C8-C20
n-Nonadecane	629-92-5	Sigma-Aldrich	Alkane standard solution C8-C20
n-Eicosane	112-95-8	Sigma-Aldrich	Alkane standard solution C8-C20

Table S3. Solute descriptors for reference compounds.

Comp	CAS-RN	*E*	*S*	*A*	*B*	*V*	*L*
2-Methylpropane	75-28-5	0.00	0.00	0.00	0.00	0.6722	1.409
n-Butane	106-97-8	0.00	0.00	0.00	0.00	0.6722	1.615
Acetone	67-64-1	0.18	0.70	0.04	0.49	0.5470	1.696
Acetonitrile	75-05-8	0.24	0.90	0.07	0.32	0.4042	1.739
Propan-2-ol	67-63-0	0.21	0.36	0.33	0.56	0.5900	1.764
2-Methylbutane	78-78-4	0.00	0.00	0.00	0.00	0.8131	2.013
Diethyl ether	60-29-7	0.04	0.25	0.00	0.45	0.7309	2.015
Dichloromethane	75-09-2	0.39	0.57	0.10	0.05	0.4943	2.019
Propan-1-ol	71-23-8	0.24	0.42	0.37	0.48	0.5900	2.031
n-Pentane	109-66-0	0.00	0.00	0.00	0.00	0.8131	2.162
Butan-2-one	78-93-3	0.17	0.70	0.00	0.51	0.6879	2.287
Ethyl acetate	141-78-6	0.11	0.62	0.00	0.45	0.7466	2.314
Carbon disulfide	75-15-0	0.88	0.26	0.00	0.03	0.4905	2.370
Trichloromethane	67-66-3	0.43	0.49	0.15	0.02	0.6167	2.480
1,2-Dichloroethane	107-06-2	0.42	0.64	0.10	0.11	0.6352	2.573
Butan-1-ol	71-36-3	0.22	0.42	0.37	0.48	0.7309	2.601
Tetrahydrofuran	109-99-9	0.29	0.52	0.00	0.48	0.6223	2.636
n-Hexane	110-54-3	0.00	0.00	0.00	0.00	0.9540	2.668
Benzene	71-43-2	0.61	0.52	0.00	0.14	0.7164	2.786
1-Nitropropane	108-03-2	0.24	0.95	0.00	0.31	0.7055	2.894
Cyclohexane	110-82-7	0.31	0.10	0.00	0.00	0.8454	2.964
Pyridine	110-86-1	0.63	0.84	0.00	0.52	0.6753	3.022
Pentan-1-ol	71-41-0	0.22	0.42	0.37	0.48	0.8718	3.106
n-Pentylamine	110-58-7	0.21	0.35	0.16	0.61	0.9129	3.139
Dimethylformamide	68-12-2	0.37	1.31	0.00	0.74	0.6468	3.173
n-Heptane	142-82-5	0.00	0.00	0.00	0.00	1.0949	3.173
Toluene	108-88-3	0.60	0.52	0.00	0.14	0.8573	3.325
n-Butyl acetate	123-86-4	0.08	0.57	0.00	0.44	1.0284	3.409
Dimethyl sulfoxide	67-68-5	0.52	1.72	0.00	0.97	0.6126	3.459
Cyclohexanol	108-93-0	0.46	0.59	0.29	0.59	0.9041	3.732
Cyclohexanone	108-94-1	0.40	0.86	0.00	0.56	0.8611	3.792
1,4-Butanediol	110-63-4	0.40	0.93	0.72	0.90	0.7896	3.795
Trimethyl phosphate	512-56-1	0.11	1.27	0.00	0.96	0.9707	3.798
Methyl phenyl ether	100-66-3	0.71	0.75	0.00	0.29	0.9160	3.890
Di-n-butyl ether	142-96-1	0.00	0.25	0.00	0.45	1.2945	3.924
Octamethyltrisiloxane (L3)	107-51-7	-0.50	-0.07	0.00	0.37	2.0903	3.936
2-Chlorophenol	95-57-8	0.88	0.67	0.54	0.34	0.8975	4.118
trans-Cyclohexane-1,2-diol	1460-57-7	0.63	0.80	0.75	0.90	0.9628	4.182
n-Nonane	111-84-2	0.00	0.00	0.00	0.00	1.3767	4.182
Octan-2-one	111-13-7	0.11	0.68	0.00	0.51	1.2515	4.257
Phenyl acetate	122-79-2	0.66	1.13	0.00	0.54	1.0726	4.414
1-Nitrohexane	646-14-0	0.20	0.95	0.00	0.29	1.1282	4.416
Octamethylcyclotetrasiloxane (D4)	556-67-2	-0.47	-0.08	0.00	0.32	2.3448	4.473
1,2-Dichlorobenzene	95-50-1	0.87	0.78	0.00	0.04	0.9612	4.518
Octan-1-ol	111-87-5	0.20	0.42	0.37	0.48	1.2945	4.619
2,6-Dimethylphenol	576-26-1	0.84	0.79	0.39	0.38	1.0569	4.680

n-Decane	124-18-5	0.00	0.00	0.00	0.00	1.5176	4.686
2-Phenylethanol	60-12-8	0.81	0.82	0.31	0.66	1.0569	4.702
Triethyl phosphate	78-40-0	0.00	1.00	0.00	1.06	1.3934	4.750
4-Chlorophenol	106-48-9	1.02	0.79	0.89	0.21	0.8975	4.802
Resorcinol	108-46-3	0.98	0.88	1.71	0.58	0.8338	4.829
1-Phenyl-1-propanol	93-54-9	0.78	0.82	0.30	0.63	1.1978	5.140
n-Undecane	1120-21-4	0.00	0.00	0.00	0.00	1.6585	5.191
Decamethylcyclopentasiloxane (D5)	541-02-6	-0.70	-0.10	0.00	0.50	2.9310	5.242
1,2,4-Trichlorobenzene	120-82-1	0.98	0.81	0.00	0.00	1.0836	5.248
Benzamide	55-21-0	1.26	1.34	0.65	0.66	0.9728	5.277
Decan-1-ol	112-30-1	0.19	0.42	0.37	0.48	1.5763	5.610
4-Nitroanisole	100-17-4	0.98	1.49	0.00	0.37	1.0902	5.620
n-Dodecane	112-40-3	0.00	0.00	0.00	0.00	1.7994	5.696
Methyl cinnamate	103-26-4	0.94	0.97	0.00	0.57	1.3114	5.890
1,3-Dinitrobenzene	99-65-0	1.15	1.60	0.00	0.47	1.0648	5.903
Dihexyl ether	112-58-3	0.00	0.25	0.00	0.45	1.8581	5.938
Dodecamethylcyclohexasiloxane (D6)	540-97-6	-0.88	-0.12	0.00	0.74	3.5172	6.080
Tri-n-propyl phosphate	513-08-6	0.05	1.00	0.00	1.15	1.8161	6.180
Dodecan-2-one	6175-49-1	0.10	0.68	0.00	0.51	1.8151	6.184
n-Tridecane	629-50-5	0.00	0.00	0.00	0.00	1.9403	6.200
Dimethyl phthalate	131-11-3	0.78	1.26	0.00	0.88	1.4288	6.275
4-Nitroaniline	100-01-6	1.24	1.83	0.60	0.34	0.9904	6.358
Dodecan-1-ol	112-53-8	0.18	0.42	0.37	0.48	1.8581	6.620
n-Tetradecane	629-59-4	0.00	0.00	0.00	0.00	2.0812	6.705
Tetradecan-2-one	2345-27-9	0.10	0.68	0.00	0.51	2.0969	7.151
n-Pentadecane	629-62-9	0.00	0.00	0.00	0.00	2.2221	7.209
n-Hexadecane	544-76-3	0.00	0.00	0.00	0.00	2.3630	7.714
n-Heptadecane	629-78-7	0.00	0.00	0.00	0.00	2.5039	8.218
n-Octadecane	593-45-3	0.00	0.00	0.00	0.00	2.6448	8.722
n-Dodecylbenzene	123-01-3	0.57	0.47	0.00	0.15	2.4072	8.855
n-Nonadecane	629-92-5	0.00	0.00	0.00	0.00	2.7857	9.226
n-Eicosane	112-95-8	0.00	0.00	0.00	0.00	2.9266	9.731

Table S4. Fixed and adjusted values of solute descriptors. The values in the table were fixed in the descriptor determination procedure. For each check mark, one value was obtained from the fitting.

	S	*A*	*B*	*V*	*L*
3:1 FTOH	✓	✓	✓	0.9948	✓
7:1 FTOH				1.8600	✓
4:2 FTOH	✓	✓	✓	1.3520	✓
6:2 FTOH				1.7846	✓
8:2 FTOH				2.2172	✓
10:2 FTOH				2.6498	✓
12:2 FTOH				3.0824	✓
3:3 FTOH	✓	✓	✓	1.2766	✓
4:4 FTOH	✓	✓	✓	1.6338	✓
5:2s FTOH	✓	✓	✓	1.5683	✓
EtFHxSE	✓	✓	✓	2.4471	✓
EtFOSE				2.8797	✓
MeFBSE				1.8736	✓
MeFOSE				2.7388	✓
PFBSA	✓	✓	✓	1.3922	✓
PFHxSA				1.8248	✓
PFOSA				2.2574	✓
MeFBSA	✓	✓	✓	1.5331	✓
MeFHxSA				1.9657	✓
MeFOSA				2.3983	✓
EtFHxSA				2.1066	✓
EtFOSA				2.5392	✓
PFBSF	✓	0	✓	1.3301	✓
PFBI	✓	0	0	1.2697	✓
PFHxI				1.7023	✓
PFHpI				1.9186	✓
PFOI				2.1349	✓
PFDI				2.5675	✓
4:2 FTI	✓	0	✓	1.5515	✓
6:2 FTI				1.9841	✓
8:2 FTI				2.4167	✓
10:2 FTI				2.8493	✓
6:1 FTI	✓	✓	✓	1.8432	✓
6:1 FTI-7H	✓	✓	✓	1.8055	✓
FE-E3	✓	✓	✓	2.7017	✓
FE-E4	✓	✓	✓	3.4093	✓
FE-E5	✓	✓	✓	4.1169	✓
AFOE	✓	0	✓	2.2397	✓
FE-E1-I	✓	0	✓	1.5447	✓
APFIPE	✓	0	✓	1.2336	✓
PFTPrA	✓	0	✓	2.2682	✓
PFTBA	✓	0	✓	2.9171	✓
PFN	✓	0	0	2.1307	✓
PFDoD				2.7796	✓
1,8-DHPFO	✓	✓	0	1.8390	✓
1,8-DVPFO	✓	0	✓	2.3166	✓

PFOSt	✓	0	✓	2.7233	✓
4:2 FTO	✓	0	0	1.2503	✓
6:2 FTO				1.6829	✓
8:2 FTO				2.1155	✓
10:2 FTO				2.5481	✓
6:2 FTAC	✓	0	✓	2.1800	✓
8:2 FTAC				2.6126	✓
10:2 FTAC				3.0452	✓
4:2 FTMAC	✓	0	✓	1.8883	✓
6:2 FTMAC				2.3209	✓
8:2 FTMAC				2.7535	✓
10:2 FTMAC				3.1861	✓
6:2 FTBnOH	✓	✓	✓	2.5333	✓
8:2 FTAce	✓	0	✓	2.5147	✓

Table S5. Selected PFAS included in the calibration sets for PP-LFER models.

3:3 FTOH
4:2 FTOH
4:4 FTOH
6:2 FTOH
7:1 FTOH
8:2 FTOH
10:2 FTOH
12:2 FTOH
5:2s FTOH
MeFBSE
MeFOSE
PFBSA
PFHxSA
PFOSA
MeFBSA
MeFHxSA
MeFOSA
PFBI
PFHxI
PFHpI
PFOI
PFDI
4:2 FTI
6:1 FTI
6:1 FTI-7H
6:2 FTI
8:2 FTI
10:2 FTI
4:2 FTO
6:2 FTO
8:2 FTO
10:2 FTO
6:2 FTAC
8:2 FTAC
10:2 FTAC
4:2 FTMAC
6:2 FTMAC
8:2 FTMAC
10:2 FTMAC

Table S6. The measured log *k* values for PFAS. NA, not available.

	HP-5ms				DB-200				DB-225				SG-WAX	
Temperature (C°)	30	50	70	90	30	60	90	120	30	60	90	120	90	120
3:1 FTOH	-0.277	-0.638	-0.912	NA	-0.184	-0.684	-1.051	NA	0.571	-0.066	-0.561	-0.961	-0.055	-0.554
3:3 FTOH	0.649	0.204	-0.161	-0.476	0.776	0.132	-0.360	-0.755	1.134	0.416	-0.146	-0.592	0.141	-0.356
4:2 FTOH	0.387	-0.030	-0.371	-0.668	0.569	-0.044	-0.513	-0.883	0.833	0.146	-0.385	-0.809	-0.045	-0.528
4:4 FTOH	1.335	0.807	0.369	-0.005	NA	0.741	0.144	-0.337	1.662	0.833	0.187	-0.326	0.391	-0.158
6:2 FTOH	0.902	0.419	0.017	-0.327	1.144	0.426	-0.130	-0.573	1.140	0.364	-0.235	-0.708	0.092	-0.449
7:1 FTOH	0.772	0.279	-0.125	-0.471	0.976	0.270	-0.273	-0.707	1.206	0.383	-0.248	-0.745	0.227	-0.367
8:2 FTOH	1.411	0.859	0.401	0.009	NA	0.893	0.251	-0.264	1.509	0.615	-0.066	-0.604	0.303	-0.336
10:2 FTOH	NA	NA	0.784	0.345	NA	NA	0.637	0.057	NA	0.921	0.143	-0.453	0.611	-0.145
12:2 FTOH	NA	NA	1.166	0.679	NA	NA	1.020	0.372	NA	1.311	0.404	NA	1.024	0.118
5:2s FTOH	0.368	-0.058	-0.402	-0.695	0.558	-0.060	-0.539	-0.913	0.862	0.126	-0.441	-0.896	-0.103	-0.610
EtFHxSE	NA	NA	NA	1.430	NA	NA	1.809	1.092	NA	NA	1.937	1.180	NA	1.413
EtFOSE	NA	NA	NA	1.760	NA	NA	2.187	1.402	NA	NA	2.089	1.278	NA	1.510
MeFBSE	NA	NA	1.407	0.934	NA	NA	1.309	0.684	NA	NA	1.719	1.028	NA	1.341
MeFOSE	NA	NA	NA	1.593	NA	NA	2.059	1.298	NA	NA	2.000	1.210	NA	1.479
PFBSA	NA	NA	0.880	0.432	NA	NA	0.652	0.110	NA	NA	NA	1.142	NA	NA
PFHxSA	NA	NA	1.263	0.765	NA	NA	1.035	0.430	NA	NA	NA	1.235	NA	NA
PFOSA	NA	NA	1.647	1.099	NA	NA	1.420	0.747	NA	NA	NA	1.341	NA	NA
MeFBSA	NA	NA	0.624	0.225	NA	NA	0.612	0.089	NA	1.856	1.108	0.508	NA	0.982
MeFHxSA	NA	NA	1.001	0.556	NA	NA	0.988	0.398	NA	NA	1.245	0.598	NA	1.036
MeFOSA	NA	NA	1.383	0.886	NA	NA	1.370	0.710	NA	NA	1.407	0.703	NA	1.127
EtFHxSA	NA	NA	1.090	0.634	NA	NA	1.103	0.493	NA	NA	1.293	0.624	NA	0.905
EtFOSA	NA	NA	1.474	0.964	NA	NA	1.484	0.803	NA	NA	1.457	0.730	NA	1.003
PFBSF	-1.041	NA	NA	NA	-0.677	-1.041	NA	NA	NA	NA	NA	NA	NA	NA
PFBI	-0.477	-0.763	-1.000	NA	-0.569	-0.986	NA	NA	-0.878	NA	NA	NA	NA	NA
PFHxI	0.042	-0.313	-0.609	-0.873	0.015	-0.503	-0.905	NA	-0.523	-1.056	NA	NA	NA	NA
PFHpI	0.296	-0.092	-0.418	-0.702	0.304	-0.266	-0.711	-1.072	-0.318	-0.915	NA	NA	NA	NA
PFOI	0.549	0.128	-0.226	-0.533	0.592	-0.030	-0.517	-0.910	-0.090	-0.757	NA	NA	NA	NA
PFDI	1.052	0.567	0.157	-0.197	1.166	0.440	-0.130	-0.587	0.424	-0.391	-0.992	NA	-0.749	NA
4:2 FTI	0.710	0.295	-0.055	-0.359	0.768	0.153	-0.331	-0.714	0.479	-0.122	-0.591	-0.954	-0.717	-1.076

6:1 FTI	0.842	0.399	0.024	-0.300	0.973	0.306	-0.219	-0.641	0.561	-0.109	-0.627	-1.029	-0.631	-1.059
6:1 FTI-7H	1.436	NA	0.491	0.128	NA	0.879	0.274	-0.211	1.809	0.974	0.322	-0.198	0.568	-0.025
6:2 FTI	NA	0.736	0.331	-0.022	1.342	0.619	0.052	-0.401	0.790	0.099	-0.436	-0.849	-0.564	-0.989
8:2 FTI	NA	1.172	0.708	0.319	NA	1.083	0.430	-0.095	1.163	0.355	-0.260	-0.741	-0.340	-0.865
10:2 FTI	NA	NA	1.093	0.650	NA	NA	0.835	0.229	1.625	0.675	-0.043	-0.593	NA	-0.630
FE-E3	-0.356	-0.747	-1.036	NA	0.205	-0.430	-0.922	NA	-0.279	-1.109	NA	NA	NA	NA
FE-E4	0.208	-0.263	-0.627	-0.947	0.850	0.088	-0.494	-0.967	0.517	-0.436	NA	NA	-0.843	NA
FE-E5	0.760	0.215	-0.236	-0.613	1.466	0.583	-0.102	-0.643	1.348	0.199	-0.660	NA	-0.237	-1.047
AFOE	1.127	0.628	0.207	-0.157	1.354	0.585	-0.015	-0.494	0.585	-0.162	-0.722	NA	-0.747	NA
FE-E1-I	-0.387	-0.698	-0.950	NA	-0.451	-0.906	NA	NA	-0.888	NA	NA	NA	NA	NA
APFIPE	NA	NA	NA	NA	-0.539	-0.999	NA	NA	-1.049	NA	NA	NA	NA	NA
PFTPrA	-0.876	NA	NA	NA	-0.453	-0.960	NA	NA	NA	NA	NA	NA	NA	NA
PFTBA	-0.206	-0.589	-0.903	NA	0.313	-0.335	-0.832	NA	-0.706	NA	NA	NA	NA	NA
PFN	-0.908	NA	NA	NA	-0.499	-0.995	NA	NA	NA	NA	NA	NA	NA	NA
PFDoD	-0.144	-0.526	-0.851	NA	0.362	-0.285	-0.784	NA	-0.624	NA	NA	NA	NA	NA
1,8-DHPFO	0.052	-0.342	-0.668	-0.944	0.570	-0.067	-0.563	-0.951	0.073	-0.572	-1.072	NA	-0.886	NA
1,8-DVPFO	1.525	0.990	0.541	0.162	NA	0.928	0.297	-0.209	1.036	0.278	-0.311	-0.773	-0.228	-0.725
PFOSt	NA	NA	NA	NA	NA	NA	1.069	0.443	NA	1.127	0.386	-0.197	-0.050	-0.182
4:2 FTO	-0.864	NA	NA	NA	-0.705	NA	NA	NA	NA	NA	NA	NA	NA	NA
6:2 FTO	-0.338	-0.662	-0.929	NA	-0.128	-0.641	-1.039	NA	-1.000	NA	NA	NA	NA	NA
8:2 FTO	0.172	-0.219	-0.546	-0.831	0.445	-0.172	-0.650	-1.033	-0.541	NA	NA	NA	NA	NA
10:2 FTO	0.678	0.222	-0.161	-0.494	1.018	0.299	-0.264	-0.714	0.005	-0.755	NA	NA	NA	NA
6:2 FTAC	1.672	1.110	0.638	0.243	NA	1.198	0.508	-0.043	1.368	0.541	-0.096	-0.599	-0.115	-0.639
8:2 FTAC	NA	NA	1.020	0.573	NA	NA	0.889	0.274	1.754	0.809	0.085	-0.477	0.144	NA
10:2 FTAC	NA	NA	1.400	0.901	NA	NA	1.270	0.586	NA	1.144	0.320	NA	0.502	NA
4:2 FTMAC	1.523	0.991	0.539	0.163	NA	0.977	0.330	-0.188	1.331	0.548	-0.061	-0.545	-0.139	-0.619
6:2 FTMAC	NA	NA	0.918	0.490	NA	NA	0.705	0.125	1.650	0.771	0.091	-0.444	0.046	NA
8:2 FTMAC	NA	NA	1.296	0.816	NA	NA	1.079	0.432	NA	1.043	0.276	-0.318	0.316	NA
10:2 FTMAC	NA	NA	1.674	1.145	NA	NA	1.459	0.742	NA	1.388	0.517	NA	0.691	NA
6:2 FTBnOH	NA	NA	NA	NA	NA	NA	NA	1.170	NA	NA	2.063	1.268	NA	1.576
8:2 FTAce	NA	NA	NA	0.329	NA	NA	0.727	0.129	1.444	0.547	-0.146	NA	NA	NA

Table S7. The measured log *k* values for reference compounds. NA, not available.

	HP-5ms				DB-200				DB-225				SG-WAX	
Temperature (C°)	30	50	70	90	30	60	90	120	30	60	90	120	90	120
2-Methylpropane	NA	NA	NA	NA	NA	NA	NA	NA	NA	NA	NA	NA	NA	NA
n-Butane	NA	NA	NA	NA	NA	NA	NA	NA	NA	NA	NA	NA	NA	NA
Acetone	-0.564	-0.792	-1.028	NA	-0.164	-0.565	-0.903	NA	-0.097	-0.501	-0.839	NA	-0.896	NA
Acetonitrile	NA	NA	NA	NA	NA	NA	NA	NA	NA	NA	NA	NA	NA	NA
Propan-2-ol	-0.542	-0.794	-1.028	NA	-0.483	-0.894	NA	NA	-0.031	-0.497	-0.886	NA	NA	NA
2-Methylbutane	-0.636	-0.858	-1.080	NA	-0.983	NA	NA	NA	NA	NA	NA	NA	NA	NA
Diethyl ether	-0.499	-0.749	-0.983	NA	-0.720	-1.007	NA	NA	-0.657	-0.995	NA	NA	NA	NA
Dichloromethane	-0.379	-0.637	-0.876	NA	-0.530	-0.889	NA	NA	-0.113	-0.504	-0.832	NA	-0.624	-0.955
Propan-1-ol	NA	-0.569	-0.818	NA	-0.288	-0.715	-1.040	NA	0.276	-0.219	-0.633	NA	-0.369	-0.748
n-Pentane	-0.533	-0.771	-1.007	NA	-0.907	NA	NA	NA	NA	NA	NA	NA	NA	NA
Butan-2-one	-0.125	-0.408	-0.683	-0.899	0.196	-0.283	-0.671	-0.970	0.261	-0.188	-0.578	-0.885	-0.670	-0.984
Ethyl acetate	-0.042	-0.356	-0.631	-0.851	0.104	-0.385	-0.776	-1.063	0.110	-0.333	-0.720	-1.022	-0.727	-1.049
Carbon disulfide	-0.365	-0.603	-0.818	-0.992	-0.739	-1.028	NA	NA	-0.408	-0.762	-1.057	NA	NA	NA
Trichloromethane	-0.033	-0.333	-0.594	-0.798	-0.292	-0.680	-0.997	NA	0.195	-0.228	-0.612	-0.890	-0.384	-0.748
1,2-Dichloroethane	0.097	-0.215	-0.485	-0.698	0.012	-0.427	-0.774	-1.025	0.440	-0.014	-0.414	-0.721	-0.253	-0.622
Butan-1-ol	0.143	-0.204	-0.493	-0.742	0.106	-0.410	-0.807	-1.097	0.660	0.114	-0.360	-0.734	-0.098	-0.521
Tetrahydrofuran	0.006	-0.326	-0.554	-0.768	0.015	-0.418	-0.780	-1.032	0.126	-0.280	-0.645	-0.915	-0.738	-1.030
n-Hexane	-0.120	-0.453	-0.670	-0.877	-0.533	-0.896	NA	NA	NA	-0.998	NA	NA	NA	NA
Benzene	0.146	-0.193	-0.438	-0.648	-0.043	-0.481	-0.814	-1.059	0.226	-0.198	-0.577	-0.859	-0.535	-0.857
1-Nitropropane	NA	0.117	-0.197	-0.453	NA	0.280	-0.178	-0.529	1.147	0.585	0.100	-0.286	NA	NA
Cyclohexane	0.143	-0.164	-0.432	-0.653	-0.271	-0.659	-0.974	NA	-0.226	-0.608	-0.927	NA	-1.083	NA
Pyridine	0.461	0.125	-0.167	-0.423	NA	0.136	-0.338	-0.680	0.934	0.408	-0.050	-0.409	0.063	-0.325
Pentan-1-ol	0.595	0.195	-0.137	-0.423	0.500	-0.075	-0.522	-0.866	1.061	0.457	-0.070	-0.481	0.185	-0.275
n-Pentylamine	NA	0.086	NA	-0.461	0.919	0.021	-0.511	-0.866	0.557	0.039	-0.435	-0.775	-0.366	NA
Dimethylformamide	0.624	0.238	-0.083	-0.365	1.530	0.763	0.198	-0.242	1.591	0.968	0.433	0.002	0.414	-0.027
n-Heptane	0.303	-0.042	-0.340	-0.590	-0.165	-0.601	-0.955	NA	-0.281	-0.685	-1.023	NA	NA	NA
Toluene	0.571	0.218	-0.094	-0.353	0.360	-0.150	-0.556	-0.844	0.625	0.128	-0.304	-0.644	-0.286	-0.635
n-Butyl acetate	0.795	0.377	0.039	-0.259	0.873	0.248	-0.245	-0.626	0.874	0.309	-0.183	-0.568	-0.244	-0.625
Dimethyl sulfoxide	NA	0.426	0.097	-0.203	NA	1.234	0.563	0.053	2.143	1.454	0.863	0.387	0.983	0.476

Cyclohexanol	1.056	0.626	0.268	-0.040	0.966	0.335	-0.152	-0.535	1.604	0.946	0.376	-0.076	0.585	0.096
Cyclohexanone	1.078	0.663	0.308	0.004	NA	0.725	0.203	-0.214	1.622	1.000	0.468	0.038	0.336	-0.072
1,4-Butanediol	NA	NA	0.469	0.115	NA	NA	0.218	-0.214	NA	NA	1.226	NA	NA	NA
Trimethyl phosphate	NA	0.824	0.433	0.084	NA	1.334	0.633	0.093	2.241	1.501	0.865	0.354	1.007	0.470
Methyl phenyl ether	NA	0.757	0.385	0.067	1.051	0.424	-0.077	-0.473	1.507	0.876	0.333	-0.104	0.451	-0.001
Di-n-butyl ether	1.072	0.634	0.261	-0.060	0.657	0.046	-0.437	-0.807	0.662	0.109	-0.375	-0.751	-0.541	-0.896
Octamethyltrisiloxane (L3)	1.111	0.642	0.247	-0.096	0.572	-0.049	-0.534	-0.901	0.206	-0.374	-0.858	NA	-1.074	NA
2-Chlorophenol	1.473	1.007	0.614	0.277	1.244	0.601	0.091	-0.322	2.408	1.635	0.967	0.435	NA	1.045
trans-Cyclohexane-1,2-diol	NA	NA	0.876	0.482	NA	NA	0.481	NA	NA	NA	1.376	0.795	NA	NA
n-Nonane	1.135	0.693	0.318	-0.007	0.567	-0.011	-0.467	-0.830	0.435	-0.083	-0.536	-0.888	-0.732	-1.038
Octan-2-one	NA	1.036	0.635	0.263	1.692	0.940	0.347	-0.129	1.727	1.039	0.447	-0.028	0.299	-0.141
Phenyl acetate	NA	1.286	0.853	0.480	NA	1.215	0.589	0.086	2.402	1.632	0.969	0.439	1.121	0.571
1-Nitrohexane	NA	1.230	0.801	0.430	NA	1.217	0.612	0.124	2.266	1.525	0.888	0.376	0.836	0.325
Octamethylcyclotetrasiloxane (D4)	NA	1.100	0.645	0.256	0.966	0.271	-0.273	-0.692	0.621	0.007	-0.541	-0.951	-0.685	NA
1,2-Dichlorobenzene	1.626	1.158	0.762	0.420	1.382	0.732	0.214	-0.204	1.837	1.184	0.624	0.171	0.793	0.314
Octan-1-ol	NA	1.340	0.884	0.493	NA	0.871	0.274	-0.201	2.217	1.431	0.751	0.210	0.985	0.418
2,6-Dimethylphenol	NA	1.441	0.992	0.607	1.738	0.996	0.409	-0.064	NA	NA	1.322	0.729	NA	1.159
n-Decane	1.557	1.066	0.650	0.291	0.936	0.298	-0.209	-0.614	0.818	0.238	-0.266	-0.664	-0.467	-0.816
2-Phenylethanol	NA	1.462	1.015	0.631	NA	1.136	0.529	0.042	NA	NA	1.353	0.771	NA	1.151
Triethyl phosphate	NA	NA	1.091	0.665	NA	NA	NA	0.513	NA	NA	1.268	0.679	NA	0.651
4-Chlorophenol	NA	NA	NA	NA	NA	NA	NA	NA	NA	NA	NA	NA	NA	NA
Resorcinol	NA	NA	1.626	1.129	NA	NA	0.938	0.384	NA	NA	NA	NA	NA	NA
1-Phenyl-1-propanol	NA	NA	1.142	0.743	NA	NA	NA	0.059	NA	NA	1.329	0.740	NA	1.097
n-Undecane	NA	NA	NA	0.581	NA	NA	0.038	NA	1.161	0.553	NA	NA	NA	-0.592
Decamethylcyclo pentasiloxane (D5)	NA	NA	1.196	0.753	NA	NA	0.202	NA	1.246	0.510	-0.131	-0.633	-0.263	-0.785
1,2,4-Trichlorobenzene	NA	1.651	1.204	0.818	NA	NA	0.541	0.080	NA	NA	0.963	0.463	NA	0.609
Benzamide	NA	NA	NA	NA	NA	NA	NA	NA	NA	NA	NA	NA	NA	NA
Decan-1-ol	NA	NA	NA	1.093	NA	NA	0.796	0.247	NA	NA	1.290	0.669	1.507	0.866
4-Nitroanisole	NA	NA	1.874	1.411	NA	NA	1.600	0.995	NA	NA	NA	1.566	NA	NA
n-Dodecane	NA	NA	NA	0.878	NA	NA	0.297	NA	1.597	0.870	0.263	NA	0.049	NA

Methyl cinnamate	NA	NA	NA	NA	NA	NA	1.356	0.749	NA	NA	NA	1.243	NA	1.459
1,3-Dinitrobenzene	NA	NA	NA	1.568	NA	NA	1.932	1.283	NA	NA	NA	NA	NA	NA
Dihexyl ether	NA	NA	1.554	1.096	NA	NA	0.579	0.049	NA	NA	0.676	0.137	NA	NA
Dodecamethylcyclohexasiloxane (D6)	NA	NA	1.795	1.285	NA	NA	0.682	0.114	1.949	1.091	0.339	-0.243	0.306	-0.352
Tri-n-propyl phosphate	NA	NA	NA	1.434	NA	NA	NA	1.064	NA	NA	NA	1.217	NA	1.101
Dodecan-2-one	NA	NA	NA	NA	NA	NA	1.376	0.745	NA	NA	1.512	0.874	NA	0.749
n-Tridecane	NA	NA	NA	1.172	NA	NA	0.554	NA	1.974	1.187	0.529	NA	0.310	NA
Dimethyl phthalate	NA	NA	NA	NA	NA	NA	1.789	1.112	NA	NA	NA	1.656	NA	NA
4-Nitroaniline	NA	NA	1.917	NA	NA	NA	NA	1.548	NA	NA	NA	NA	NA	NA
Dodecan-1-ol	NA	NA	NA	NA	NA	NA	NA	NA	NA	NA	1.819	NA	NA	NA
n-Tetradecane	NA	NA	NA	1.465	NA	NA	0.808	0.239	2.326	1.501	0.794	0.223	0.571	0.062
Tetradecan-2-one	NA	NA	NA	NA	NA	NA	NA	NA	NA	NA	NA	1.314	NA	1.182
n-Pentadecane	NA	NA	NA	1.757	NA	NA	1.060	0.453	2.697	1.815	1.057	0.447	0.831	0.286
n-Hexadecane	NA	NA	NA	NA	NA	NA	1.311	NA	NA	NA	1.319	0.670	1.090	0.507
n-Heptadecane	NA	NA	NA	NA	NA	NA	1.561	NA	NA	NA	1.579	0.890	1.347	0.726
n-Octadecane	NA	NA	NA	NA	NA	NA	NA	NA	NA	NA	1.837	1.104	1.603	0.944
n-Dodecylbenzene	NA	NA	NA	NA	NA	NA	NA	1.389	NA	NA	NA	1.709	NA	1.668
n-Nonadecane	NA	NA	NA	NA	NA	NA	NA	NA	NA	NA	NA	1.326	NA	1.160
n-Eicosane	NA	NA	NA	NA	NA	NA	NA	NA	NA	NA	NA	1.542	NA	NA

Table S8. Final adjusted solute descriptors for PFAS.

	Value					Standard error				SD	*n**
	S	*A*	*B*	*V*	*L*	*S*	*A*	*B*	*L*		
3:1 FTOH	0.30	0.84	0.14	0.995	1.542	0.02	0.02	0.01	0.004	0.07	25
3:3 FTOH	0.53	0.52	0.39	1.277	2.710	0.01	0.02	0.01	0.010	0.04	14
4:2 FTOH	0.35	0.60	0.31	1.352	2.421	0.02	0.01	0.01	0.038	0.09	57
4:4 FTOH	0.52	0.51	0.44	1.634	3.499	0.03	0.03	0.01	0.013	0.06	13
6:2 FTOH	0.35	0.60	0.31	1.785	2.997	0.02	0.01	0.01	0.023	0.09	57
7:1 FTOH	0.30	0.84	0.14	1.860	2.715	0.02	0.02	0.01	NA	0.07	25
8:2 FTOH	0.35	0.60	0.31	2.217	3.554	0.02	0.01	0.01	0.020	0.09	57
10:2 FTOH	0.35	0.60	0.31	2.650	4.117	0.02	0.01	0.01	0.015	0.09	57
12:2 FTOH	0.35	0.60	0.31	3.082	4.682	0.02	0.01	0.01	0.045	0.09	57
5:2s FTOH	0.39	0.62	0.19	1.568	2.296	0.02	0.02	0.01	0.140	0.05	14
EtFHxSE	1.00	0.55	0.68	2.447	5.685	0.02	0.03	0.02	NA	0.08	24
EtFOSE	1.00	0.55	0.68	2.880	6.253	0.02	0.03	0.02	NA	0.08	24
MeFBSE	1.00	0.55	0.68	1.874	4.769	0.02	0.03	0.02	NA	0.08	24
MeFOSE	1.00	0.55	0.68	2.739	5.909	0.02	0.03	0.02	NA	0.08	24
PFBSA	0.96	1.15	0.23	1.392	3.607	0.03	0.03	0.02	0.051	0.08	19
PFHxSA	0.96	1.15	0.23	1.825	4.189	0.03	0.03	0.02	0.033	0.08	19
PFOSA	0.96	1.15	0.23	2.257	4.744	0.03	0.03	0.02	NA	0.08	19
MeFBSA	0.88	0.74	0.24	1.533	3.566	0.02	0.02	0.01	0.028	0.07	35
MeFHxSA	0.88	0.74	0.24	1.966	4.135	0.02	0.02	0.01	0.004	0.07	35
MeFOSA	0.88	0.74	0.24	2.398	4.703	0.02	0.02	0.01	0.015	0.07	35
EtFHxSA	0.88	0.74	0.24	2.107	4.400	0.02	0.02	0.01	0.012	0.07	35
EtFOSA	0.88	0.74	0.24	2.539	4.967	0.02	0.02	0.01	0.030	0.07	35
PFBSF	0.16	0	NA	1.330	1.278	NA	NA	NA	NA	0.09	3
PFBI	0.07	0	0	1.270	1.939	0.01	NA	NA	0.006	0.04	46
PFHxI	0.07	0	0	1.702	2.508	0.01	NA	NA	0.007	0.04	46
PFHpI	0.07	0	0	1.919	2.792	0.01	NA	NA	0.016	0.04	46
PFOI	0.07	0	0	2.135	3.068	0.01	NA	NA	0.008	0.04	46
PFDI	0.07	0	0	2.568	3.623	0.01	NA	NA	0.007	0.04	46
4:2 FTI	0.32	0.00	0.17	1.552	3.325	0.01	NA	0.01	0.012	0.05	42
6:1 FTI	0.27	0.13	0.11	1.843	3.382	0.02	0.02	0.01	0.016	0.04	14
6:1 FTI-7H	0.63	0.24	0.14	1.806	3.887	0.04	0.04	0.02	0.009	0.09	12
6:2 FTI	0.32	0	0.17	1.984	3.892	0.01	NA	0.01	0.006	0.05	42
8:2 FTI	0.32	0	0.17	2.417	4.455	0.01	NA	0.01	0.002	0.05	42
10:2 FTI	0.32	0	0.17	2.849	5.020	0.01	NA	0.01	0.023	0.05	42
FE-E3	-0.33	0.68	NA	2.702	1.899	0.17	0.24	NA	0.075	0.28	8
FE-E4	-0.55	1.03	NA	3.409	2.542	0.21	0.30	NA	0.091	0.38	10
FE-E5	-0.81	1.55	NA	4.117	3.128	0.27	0.37	NA	0.262	0.48	11
AFOE	0.30	0	NA	2.240	3.584	NA	NA	NA	0.014	0.03	11
FE-E1-I	0.06	0	NA	1.545	1.937	NA	NA	NA	0.016	0.08	6
APFIPE	0.15	0	NA	1.234	1.717	NA	NA	NA	0.014	0.03	3
PFTPrA	-0.26	0	NA	2.268	1.521	NA	NA	NA	0.046	0.15	3
PFTBA	-0.23	0	NA	2.917	2.302	NA	NA	NA	0.028	0.26	7

	Value					Standard error				SD	*n*
	S	*A*	*B*	*V*	*L*	*S*	*A*	*B*	*L*		
PFN	-0.19	0	0	2.131	1.571	0.06	NA	NA	0.013	0.21	10
PFDoD	-0.19	0	0	2.780	2.362	0.06	NA	NA	0.050	0.21	10
1,8-DHPFO	0.38	0.24	0	1.839	2.130	0.02	0.03	NA	0.039	0.03	11
1,8-DVPFO	0.44	0	NA	2.317	3.927	NA	NA	NA	0.017	0.05	11
PFOSt	0.32	0	NA	2.723	5.645	NA	NA	NA	0.028	0.04	5
4:2 FTO	0.11	0	0	1.250	1.451	0.01	NA	NA	0.014	0.04	28
6:2 FTO	0.11	0	0	1.683	2.022	0.01	NA	NA	0.023	0.04	28
8:2 FTO	0.11	0	0	2.116	2.504	0.01	NA	NA	0.025	0.04	28
10:2 FTO	0.11	0	0	2.548	3.031	0.01	NA	NA	NA	0.04	28
6:2 FTAC	0.54	0	0.40	2.180	4.098	0.01	NA	0.01	0.031	0.04	26
8:2 FTAC	0.54	0	0.40	2.613	4.661	0.01	NA	0.01	0.009	0.04	26
10:2 FTAC	0.54	0	0.40	3.045	5.221	0.01	NA	0.01	0.009	0.04	26
4:2 FTMAC	0.53	0	0.39	1.888	3.990	0.01	NA	0.01	0.043	0.05	33
6:2 FTMAC	0.53	0	0.39	2.321	4.553	0.01	NA	0.01	0.020	0.05	33
8:2 FTMAC	0.53	0	0.39	2.754	5.115	0.01	NA	0.01	0.001	0.05	33
10:2 FTMAC	0.53	0	0.39	3.186	5.671	0.01	NA	0.01	0.018	0.05	33
6:2 FTBnOH	0.75	0.41	NA	2.533	6.656	0.05	0.07	NA	NA	0.04	3
8:2 FTAce	0.54	0	NA	2.515	4.234	NA	NA	NA	0.025	0.05	6

**n* indicates the number of data on which the determined *S*, *A*, and *B* descriptors are based. Note that for a group of PFAS (e.g., X:2 FTOHs), the *S*, *A*, and *B* values were assumed to be common and determined simultaneously. Thus, *n* indicates the “total number” of data for the group of PFAS.

Table S9. PP-LFER system parameters obtained in this study.

	Value						SE						R^2	SD	n
	c	*s*	*a*	*b*	*v*	*l*	*c*	*s*	*a*	*b*	*v*	*l*			
SQ 75m 30°C	-2.498	0.111	0	0	0.045	0.932	0.015	0.023	NA	NA	0.008	0.008	0.999	0.017	20
SQ 10m 30°C	-2.299	0.094	0	0	0	0.906	0.024	0.026	NA	NA	NA	0.007	0.999	0.025	21
SPB-Octyl 30°C	-2.396	0.143	0.258	0	0	0.878	0.030	0.024	0.030	NA	NA	0.010	0.996	0.041	41
SPB-Octyl 70°C	-2.481	0.046	0.124	0	-0.141	0.753	0.023	0.018	0.015	NA	0.009	0.008	0.997	0.032	53
SPB-Octyl 100°C	-2.375	0.055	0	0	-0.206	0.637	0.035	0.016	NA	NA	0.010	0.007	0.994	0.045	63
Apolane 87 30°C	-2.892	0	0.104	0	-0.153	0.994	0.070	NA	0.055	NA	0.025	0.016	0.994	0.054	30
Apolane 87 40°C	-3.244	0.049	0	0	-0.087	0.930	0.061	0.031	NA	NA	0.028	0.016	0.993	0.052	32
Log $K_{\mathrm{Hxd/air}}$ 25°C	0	0	0	0	0	1	NA	NA	NA	NA	NA	1	NA	NA	NA
HP-5ms 30°C	-2.358	0.526	0.539	0	0.316	0.726	0.026	0.029	0.029	NA	0.014	0.009	0.996	0.043	50
HP-5ms 50°C	-2.382	0.440	0.435	0	0.222	0.660	0.018	0.019	0.021	NA	0.010	0.006	0.998	0.035	62
HP-5ms 70°C	-2.437	0.407	0.376	0	0.155	0.604	0.018	0.019	0.015	NA	0.009	0.007	0.997	0.045	86
HP-5ms 90°C	-2.462	0.352	0.299	0	0.092	0.556	0.018	0.014	0.013	NA	0.008	0.005	0.997	0.040	86
DB-200 30°C	-2.525	1.514	0.511	0	0.762	0.462	0.067	0.083	0.077	NA	0.038	0.021	0.978	0.110	49
DB-200 60°C	-2.625	1.360	0.366	0	0.604	0.406	0.053	0.052	0.056	NA	0.028	0.017	0.981	0.094	60
DB-200 90°C	-2.704	1.154	0.261	0	0.449	0.378	0.034	0.027	0.026	NA	0.016	0.008	0.990	0.081	89
DB-200 120°C	-2.703	1.020	0.188	0	0.359	0.318	0.034	0.028	0.024	NA	0.015	0.008	0.989	0.073	80
SolGel-WAX 90°C	-2.715	1.414	1.777	0	-0.181	0.551	0.064	0.054	0.067	NA	0.025	0.014	0.976	0.107	61
SolGel-WAX 120°C	-2.772	1.251	1.629	0	-0.356	0.538	0.044	0.038	0.049	NA	0.025	0.010	0.989	0.087	66
DB-225ms 30°C	-2.551	1.616	1.635	0	0.057	0.709	0.037	0.034	0.043	NA	0.018	0.009	0.994	0.071	66
DB-225ms 60°C	-2.560	1.360	1.342	0	-0.136	0.648	0.030	0.026	0.034	NA	0.014	0.009	0.994	0.061	70
DB-225ms 90°C	-2.629	1.202	1.129	0	-0.240	0.588	0.034	0.029	0.035	NA	0.016	0.008	0.992	0.076	81
DB-225ms 120°C	-2.697	1.108	0.981	0	-0.294	0.528	0.038	0.030	0.032	NA	0.020	0.007	0.991	0.078	76
Log K_{ow} 25°C	0.331	-1.412	-0.137	-3.434	2.447	0.425	0.029	0.042	0.039	0.041	0.043	0.013	0.990	0.144	328
Log K_{aw} 25°C	0.525	-2.065	-3.634	-4.862	2.620	-0.484	0.023	0.033	0.032	0.032	0.033	0.010	0.996	0.131	410
Log K_{oa} 25°C	-0.152	0.443	3.481	0.874	0.100	0.902	0.031	0.052	0.076	0.058	0.061	0.017	0.997	0.154	180
Log $K_{\mathrm{Hxd/w}}$ 25°C	0.529	-1.973	-3.617	-4.915	2.773	0.464	0.034	0.052	0.055	0.054	0.053	0.017	0.991	0.176	296

Table S10. Log *K* values of **neutral (species of) PFAS** predicted by COSMO*therm* (25°C, unless otherwise noted).

Name	Abbreviation	CAS-RN	log K_{ow}	log K_{aw}	log $K_{oa,dry}$	log $K_{oa,wet}$	log $K_{Hxd/air}$	log $K_{oil/w}$ 37°C	log $K_{oil/air}$ 37°C
1H,1H-Perfluorobutan-1-ol	3:1 FTOH	375-01-9	2.30	-1.83	4.08	4.13	1.84	1.74	3.28
3-(Perfluoropropyl)propan-1-ol	3:3 FTOH	679-02-7	2.70	-2.39	5.04	5.09	3.06	2.15	4.16
1H,1H,2H,2H-Perfluorohexan-1-ol	4:2 FTOH	2043-47-2	3.00	-1.99	4.97	4.99	2.94	2.48	4.11
4-(Perfluorobutyl)butan-1-ol	4:4 FTOH	3792-02-7	3.68	-2.22	5.86	5.90	3.93	3.06	4.85
1H,1H,2H,2H-Perfluorooctan-1-ol	6:2 FTOH	647-42-7	4.37	-1.23	5.62	5.59	3.65	3.87	4.70
1H,1H-Perfluorooctan-1-ol	7:1 FTOH	307-30-2	4.97	-0.36	5.38	5.33	3.25	4.47	4.47
1H,1H,2H,2H-Perfluorodecan-1-ol	8:2 FTOH	678-39-7	5.63	-0.36	6.07	6.00	4.27	5.16	5.11
1H,1H,2H,2H-Perfluorododecan-1-ol	10:2 FTOH	865-86-1	7.06	0.44	6.76	6.62	4.87	6.64	5.74
1H,1H,2H,2H-Perfluorotetradecan-1-ol	12:2 FTOH	39239-77-5	8.38	1.26	7.30	7.12	5.45	7.99	6.23
1H,1H,2H,2H-Perfluorobutan-1-ol	2:2 FTOH	54949-74-5	1.92	-2.26	4.11	4.18	1.94	1.34	3.29
1H,1H,1H,2H-Perfluoroheptan-2-ol	5:2s FTOH	914637-05-1	4.01	-0.87	4.89	4.88	2.96	3.46	3.99
1H,1H,1H,2H-Perfluorononan-2-ol	7:2s FTOH	24015-83-6	5.33	-0.13	5.53	5.46	3.64	4.81	4.56
3-(Perfluoro-2-butyl)propane-1,2-diol	NFHp-1,2-diol	125070-38-4	2.40	-4.51	6.74	6.91	3.98	1.55	5.54
Pentafluoropropanoic anhydride	PFPrAnhy	356-42-3	4.68	2.89	1.88	1.80	1.75	4.74	1.70
Perfluoroheptane	PFHp	335-57-9	6.03	4.93	1.30	1.10	1.68	5.95	0.91
Perfluorooctane	PFO	307-34-6	6.73	5.39	1.56	1.34	2.00	6.68	1.17
Perfluorononane	PFN	375-96-2	7.38	5.79	1.85	1.60	2.34	7.36	1.44
perfluorodecane	PFD	307-45-9	8.08	6.23	2.13	1.85	2.69	8.08	1.70
perfluoroundacane	PFUnD	307-49-3	8.73	6.65	2.39	2.08	3.01	8.77	1.96
Perfluorododecane	PFDoD	307-59-5	9.41	7.12	2.63	2.29	3.30	9.47	2.18
Perfluorobutanesulfonyl fluoride	PFBSF	375-72-4	4.64	2.58	2.19	2.06	2.34	4.54	1.81
Perfluorohexanesulfonyl fluoride	PFHxSF	423-50-7	6.05	3.50	2.74	2.55	3.02	6.00	2.33
Perfluorooctanesulfonyl fluoride	PFOSF	307-35-7	7.43	4.43	3.25	3.00	3.66	7.45	2.82
Perfluorobutyl iodide	PFBI	423-39-2	4.45	2.23	2.35	2.22	2.48	4.34	1.93
Perfluorohexyl iodide	PFHxI	355-43-1	5.87	3.14	2.92	2.73	3.17	5.82	2.47
Perfluoroheptyl iodide	PFHpI	335-58-0	6.55	3.57	3.20	2.98	3.51	6.53	2.74
Perfluorooctyl iodide	PFOI	507-63-1	7.23	4.02	3.46	3.21	3.83	7.24	2.99
Perfluorodecyl iodide	PFDI	423-62-1	8.63	4.94	4.00	3.69	4.49	8.71	3.51
1,8-Diiodoperfluorooctane	1,8-DIPFO	335-70-6	7.71	2.62	5.35	5.08	5.65	7.79	4.82
1H,1H,2H,2H-Perfluorohexyl iodide	4:2 FTI	2043-55-2	4.79	0.83	4.09	3.96	3.96	4.84	3.75
1H,1H-Perfluoroheptyl iodide	6:1 FTI	212563-43-4	5.86	1.83	4.19	4.03	4.02	5.94	3.84

1H,1H,7H-Perfluoroheptyl iodide	6:1 FTI-7H	376-32-9	5.26	0.39	4.95	4.87	4.41	5.44	4.73
1H,1H,2H,2H-Perfluorooctyl iodide	6:2 FTI	2043-57-4	6.21	1.78	4.63	4.44	4.63	6.33	4.27
1H,1H,2H,2H-Perfluorodecyl iodide	8:2 FTI	2043-53-0	7.56	2.69	5.11	4.86	5.23	7.74	4.73
1H,1H,2H,2H-Perfluorododecyl iodide	10:2 FTI	2043-54-1	8.96	3.68	5.59	5.28	5.84	9.21	5.19
1,1,1,2,2,3,3-Heptafluoro-3-[(1,1,1,2,3,3-hexafluoro-3-{[1,1,1,2,3,3-hexafluoro-3-(1,2,2,2-tetrafluoroethoxy)-2-propanyl]oxy}-2-propanyl)oxy]propane	FE-E3	3330-16-3	8.91	6.58	2.61	2.33	2.66	9.12	2.36
1,1,1,2,4,4,5,7,7,8,10,10,11,13,13,14,14,15,15,15-Eicosafluoro-5,8,11-tris(trifluoromethyl)-3,6,9,12-tetraoxapentadecane	FE-E4	26738-51-2	11.02	8.24	3.15	2.78	3.36	11.33	2.88
1,1,1,2,4,4,5,7,7,8,10,10,11,13,13,14,16,16,17,17,18,18,18-Tricosafluoro-5,8,11,14-tetrakis(trifluoromethyl)-3,6,9,12,15-pentaoxaoctadecane	FE-E5	37486-69-4	13.07	9.70	3.83	3.37	4.21	13.49	3.54
Allyl 1H,1H-perfluorooctyl ether	AFOE	812-72-6	6.73	2.63	4.27	4.10	4.18	6.85	3.94
1-(Heptafluoropropoxy)-1,2,2,2-tetrafluoro-1-iodoethane	FE-E1-I	107432-46-2	5.43	3.35	2.25	2.08	2.46	5.37	1.84
Allyl perfluoroisopropyl ether	APFIPE	15242-17-8	4.12	1.99	2.22	2.13	2.11	4.07	1.93
Perfluorotripropyl amine	PFTPrA	338-83-0	7.34	5.51	2.08	1.83	2.57	7.30	1.65
Perfluorotributyl amine	PFTBA	311-89-7	9.40	7.00	2.73	2.39	3.40	9.46	2.28
1H,8H-Perfluorooctane	1,8-DHPFO	307-99-3	5.69	2.51	3.28	3.18	2.80	5.84	3.14
1,8-Divinylperfluorooctane	1,8-DVPFO	35192-44-0	7.09	2.68	4.62	4.41	4.46	7.27	4.34
4-(Perfluorooct-1-yl)styrene	PFOSt	106209-21-6	8.83	3.05	6.06	5.78	6.25	9.01	5.64
1H,1H,2H-Perfluoro-1-hexene	4:2 FTO	19430-93-4	4.19	2.32	1.98	1.87	1.89	4.13	1.68
1H,1H,2H-Perfluoro-1-octene	6:2 FTO	25291-17-2	5.56	3.15	2.57	2.40	2.59	5.55	2.25
1H,1H,2H-Perfluoro-1-decene	8:2 FTO	21652-58-4	6.90	4.11	3.02	2.80	3.14	6.97	2.69
1H,1H,2H-Perfluoro-1-dodecene	10:2 FTO	30389-25-4	8.22	4.86	3.64	3.36	3.89	8.35	3.28
1H,1H,2H,2H-Perfluorohexyl acrylate	4:2 FTAC	52591-27-2	4.35	0.14	4.24	4.21	3.87	4.35	3.89
1H,1H,2H,2H-Perfluorooctyl acrylate	6:2 FTAC	17527-29-6	5.71	1.00	4.81	4.72	4.54	5.77	4.43
1H,1H,2H,2H-Perfluorodecyl acrylate	8:2 FTAC	27905-45-9	7.02	2.00	5.17	5.02	5.04	7.15	4.78
1H,1H,2H,2H-Perfluorododecyl acrylate	10:2 FTAC	17741-60-5	8.56	3.04	5.74	5.52	5.76	8.76	5.34
1H,1H,2H,2H-Perfluorohexyl methacrylate	4:2 FTMAC	1799-84-4	4.89	0.35	4.59	4.53	4.30	4.90	4.20
1H,1H,2H,2H-Perfluorooctyl methacrylate	6:2 FTMAC	2144-53-8	6.14	1.07	5.18	5.07	4.96	6.21	4.75
1H,1H,2H,2H-Perfluorodecyl methacrylate	8:2 FTMAC	1996-88-9	7.34	1.79	5.69	5.55	5.52	7.45	5.24
1H,1H,2H,2H-Perfluorododecyl methacrylate	10:2 FTMAC	2144-54-9	8.78	3.08	5.93	5.70	5.95	8.99	5.48
4-(1H,1H,2H,2H-Perfluorooctyl)benzyl alcohol	6:2 FTBnOH	356055-76-0	6.21	-2.54	8.73	8.75	6.76	5.57	7.52
1H,1H,2H,2H-Perfluorodecyl acetate	8:2 FTAce	37858-04-1	6.37	1.37	5.10	5.00	4.83	6.41	4.67

Perfluorobutane sulfonamide	PFBSA	30334-69-1	2.96	-4.95	7.88	7.91	3.97	2.20	6.65
Perfluorohexane sulfonamide	PFHxSA	41997-13-1	4.38	-4.20	8.61	8.58	4.65	3.59	7.26
Perfluorooctane sulfonamide	PFOSA	754-91-6	5.71	-3.44	9.24	9.15	5.33	4.96	7.84
N-Methyl perfluorobutane sulfonamide	MeFBSA	68298-12-4	3.48	-2.88	6.36	6.35	4.18	3.13	5.60
N-Methyl perfluorohexane sulfonamide	MeFHxSA	68259-15-4	4.83	-2.04	6.94	6.88	4.82	4.50	6.11
N-Methyl perfluorooctane sulfonamide	MeFOSA	31506-32-8	6.17	-1.20	7.50	7.38	5.46	5.91	6.65
N-Ethyl perfluorobutane sulfonamide	EtFBSA	40630-67-9	4.88	-3.69	8.56	8.56	6.28	4.50	7.57
N-Ethyl perfluorohexane sulfonamide	EtFHxSA	87988-56-5	5.35	-1.75	7.17	7.10	5.21	5.04	6.33
N-Ethyl perfluorooctane sulfonamide	EtFOSA	4151-50-2	6.71	-0.92	7.77	7.63	5.87	6.45	6.88
N-Methyl perfluorobutane sulfonamidoethanol	MeFBSE	34454-97-2	2.87	-4.76	7.52	7.63	5.22	2.45	6.66
N-Methyl perfluorohexane sulfonamidoethanol	MeFHxSE	68555-75-9	4.22	-3.97	8.13	8.19	5.82	3.83	7.22
N-Methyl perfluorooctane sulfonamidoethanol	MeFOSE	24448-09-7	5.57	-3.11	8.72	8.69	6.56	5.30	7.80
N-Ethyl perfluorobutane sulfonamidoethanol	EtFBSE	34449-89-3	3.60	-4.43	7.99	8.03	5.73	3.25	7.11
N-Ethyl perfluorohexane sulfonamidoethanol	EtFHxSE	34455-03-3	4.74	-3.98	8.71	8.72	6.34	4.45	7.81
N-Ethyl perfluorooctane sulfonamidoethanol	EtFOSE	1691-99-2	5.98	-3.15	9.13	9.13	7.19	5.61	8.11
N,N-Dimethyl perfluorooctane sulfonamide	DiMeFOSA	213181-78-3	6.51	0.93	5.72	5.59	5.48	6.71	5.42
2,2,3,3,4,4-hexafluoropentane-1,5-diol		376-90-9	1.01	-6.74	7.47	7.76	3.35	-0.07	6.14
Perfluoro-1-octanol		114292-89-6	6.62	-0.44	7.29	7.06	3.24	6.07	6.13
5H-Octafluoropentanoyl fluoride		813-03-6	3.73	1.73	2.05	2.00	1.65	3.72	1.85
Octafluorobutan-1-one		335-42-2	3.50	2.71	0.86	0.78	0.89	3.37	0.56
Perfluorobutanal		375-02-0	2.84	1.17	1.70	1.67	1.34	2.76	1.45
Perfluoroglutaryl fluoride		678-78-4	3.80	2.23	1.65	1.57	1.55	3.72	1.37
[4-(Heptadecafluorooctyl)phenyl]methanol		163114-33-8	7.28	-0.92	8.35	8.20	6.39	6.90	7.30
4-(Heptadecafluorooctyl)phenol		80804-82-6	7.72	-1.26	9.18	8.98	6.32	7.26	8.03
(Perfluoro-5-methylhexyl)ethyl 2-methylprop-2-enoate		50836-66-3	6.70	1.29	5.54	5.41	5.39	6.79	5.10
1H-Perfluoro-1,1-propanediol		422-63-9	1.40	-5.11	6.36	6.51	2.19	0.53	5.21
1H,1H-Perfluorooctyl acrylate		307-98-2	6.40	2.11	4.42	4.29	4.28	6.51	4.10
Hexafluoroglutaryl chloride		678-77-3	4.49	1.47	3.13	3.02	3.13	4.42	2.74
(Heptafluoropropyl)trimethylsilane		3834-42-2	4.57	2.31	2.37	2.26	2.36	4.52	2.05
Bis(1H,1H-perfluoropropyl)amine		883498-76-8	4.12	0.91	3.28	3.22	2.58	4.17	3.03
Perfluoro-1-octanesulfonyl chloride		423-60-9	7.65	3.96	3.94	3.69	4.34	7.69	3.48
Methyl perfluoro(3-(1-ethenyloxypropan-2-yloxy)propanoate)		63863-43-4	6.03	3.04	3.13	2.99	3.03	6.14	2.89
2,2,3,3,4,4,4-Heptafluorobutyl methacrylate		13695-31-3	4.16	0.63	3.57	3.53	3.33	4.13	3.23

(Heptafluorobutanoyl)pivaloylmethane	17587-22-3	3.74	-1.01	4.72	4.75	4.01	3.79	4.42
Perfluorocyclohexanecarbonyl fluoride	6588-63-2	4.67	2.85	1.95	1.82	2.11	4.56	1.57
2-(Perfluorooctyl)ethanthiol	34143-74-3	7.07	2.66	4.65	4.41	4.59	7.24	4.30
1H,1H-Perfluoro-3,6,9-trioxadecan-1-ol	147492-57-7	5.55	1.10	4.52	4.44	2.63	5.14	3.70
1H,1H,7H-Perfluoroheptyl 4-methylbenzenesulfonate	424-16-8	6.16	-2.54	8.68	8.70	7.84	6.61	8.59
3-(Perfluorooctyl)propanol	1651-41-8	5.96	-0.43	6.48	6.39	4.60	5.50	5.47
1H,1H,8H,8H-Perfluoro-3,6-dioxaoctane-1,8-diol	129301-42-4	2.63	-5.57	8.01	8.20	3.42	1.66	6.68
1H,1H,7H-Dodecafluoro-1-heptanol	335-99-9	3.77	-2.11	5.82	5.88	3.30	3.33	5.06
1H,1H,5H-Perfluoropentanol	355-80-6	2.35	-2.74	4.97	5.09	2.57	1.87	4.26
Methyl 2H,2H,3H,3H-perfluoroheptanoate	132424-36-3	3.89	-0.10	4.01	3.99	3.67	3.86	3.66
1H,1H,11H,11H-Perfluorotetraethylene glycol	330562-44-2	4.49	-4.17	8.56	8.66	3.89	3.55	7.14
1H,1H,8H,8H-Perfluorooctane-1,8-diol	90177-96-1	3.04	-6.05	8.89	9.08	4.46	1.99	7.45
2-Aminohexafluoropropan-2-ol	31253-34-6	1.61	-3.41	4.85	5.02	2.07	0.77	3.83
1-Iodo-1H,1H,2H,2H-perfluoroheptane	1682-31-1	5.51	1.29	4.39	4.22	4.33	5.59	4.03
1-Bromopentadecafluoroheptane	375-88-2	6.39	4.06	2.54	2.33	2.90	6.33	2.10
1-(Perfluorohexyl)octane	133331-77-8	8.96	4.06	5.20	4.89	5.59	9.11	4.74
Dimethoxymethyl((perfluorohexyl)ethyl)silane	85857-17-6	6.23	1.68	4.68	4.55	4.56	6.27	4.16
Triethoxy((perfluorohexyl)ethyl)silane	51851-37-7	7.55	1.72	6.00	5.82	6.05	7.79	5.48
2,2,2-Trifluoroethyl perfluorobutanesulfonate	79963-95-4	5.39	1.46	4.03	3.93	3.56	5.52	3.81
(Perfluorobutyryl)-2-thenoylmethane	559-94-4	3.94	-2.70	6.59	6.64	5.65	4.01	6.27
tris(Trifluoroethoxy)methane	58244-27-2	4.35	0.57	3.79	3.78	3.00	4.60	3.78
Trichloro((perfluorohexyl)ethyl)silane	78560-45-9	7.51	2.93	4.84	4.58	4.89	7.74	4.47
Heptafluorobutyl iodide	374-98-1	3.76	0.49	3.35	3.27	3.03	3.75	3.03
2-(Trifluoromethoxy)ethyl trifluoromethanesulfonate	329710-76-1	3.46	-1.20	4.71	4.66	3.81	3.63	4.53
1H,1H,5H,5H-Perfluoro-1,5-pentanediol diacrylate	678-95-5	3.92	-2.58	6.39	6.50	5.71	4.00	6.12
Dichloromethyl((perfluorohexyl)ethyl)silane	73609-36-6	7.20	2.78	4.64	4.42	4.60	7.44	4.35
3H-Perfluoro-2,2,4,4-tetrahydroxypentane	77953-71-0	2.07	-8.52	10.38	10.59	4.42	0.83	8.80
1H,1H,9H-Perfluorononyl acrylate	4180-26-1	6.47	1.05	5.50	5.42	5.00	6.70	5.29
2,2-Difluoroethyl triflate	74427-22-8	2.60	-1.28	3.85	3.88	3.14	2.66	3.71
3H,3H-Perfluoro-2,4-hexanedione	20825-07-4	3.47	0.67	2.79	2.80	2.04	3.55	2.67
1H,1H,10H,10H-Perfluorodecane-1,10-diol	754-96-1	4.44	-5.33	9.64	9.76	5.02	3.45	8.15
2,2,3,3-Tetrafluoropropyl acrylate	7383-71-3	2.38	-1.20	3.47	3.58	2.88	2.34	3.28
1H,1H,6H,6H-Perfluorohexane-1,6-diol diacrylate	2264-01-9	4.63	-1.94	6.51	6.57	5.84	4.77	6.26

Perfluorooct-1-ene		559-14-8	6.20	4.54	1.86	1.66	2.14	6.16	1.48
6:1 Fluorotelomer alcohol		375-82-6	4.34	-0.67	5.05	5.02	2.87	3.83	4.16
3-(Perfluoro-3-methylbutyl)-1,2-propenoxide		54009-81-3	4.01	0.38	3.68	3.63	3.38	3.98	3.34
1H,1H-Heptafluorobutyl epoxide		1765-92-0	2.84	-0.17	3.01	3.01	2.61	2.77	2.71
Ethyl pentafluoropropionyl acetate		663-35-4	2.81	-1.03	3.76	3.84	3.19	2.78	3.50
3,3-Bis(trifluoromethyl)-2-propenoic acid		1763-28-6	2.69	-3.67	6.33	6.36	2.74	2.23	5.53
3-(Perfluorohexyl)-1,2-epoxypropane		38565-52-5	4.82	1.09	3.81	3.73	3.55	4.84	3.48
N-Methyl-N-trimethylsilylheptafluorobutyramide		53296-64-3	4.53	0.37	4.19	4.17	3.82	4.47	3.78
Trifluoroacetic acid	TFA	76-05-1	1.57	-3.77	5.33	5.34	1.44	1.03	4.49
Perfluoropropionic acid	PFPrA	422-64-0	2.32	-3.35	5.69	5.67	1.71	1.80	4.82
Perfluorobutanoic acid	PFBA	375-22-4	3.07	-2.93	6.05	6.00	2.07	2.55	5.14
Perfluoropentanoic acid	PFPeA	2706-90-3	3.75	-2.55	6.38	6.30	2.40	3.23	5.42
Perfluorohexanoic acid	PFHxA	307-24-4	4.46	-2.12	6.69	6.58	2.75	3.95	5.71
Perfluoroheptanoic acid	PFHpA	375-85-9	5.16	-1.79	7.09	6.95	3.17	4.67	6.07
Perfluorooctanoic acid	PFOA	335-67-1	5.83	-1.41	7.41	7.24	3.49	5.35	6.36
Perfluorononanoic acid	PFNA	375-95-1	6.57	-0.97	7.73	7.53	3.84	6.11	6.66
Perfluorodecanoic acid	PFDA	335-76-2	7.28	-0.53	8.04	7.80	4.15	6.85	6.94
Perfluoroundecanoic acid	PFUnDA	2058-94-8	7.81	-0.15	8.22	7.97	4.37	7.37	7.08
Perfluorododecanoic acid	PFDoDA	307-55-1	8.44	0.31	8.41	8.13	4.63	8.01	7.25
Perfluorotridecanoic acid	PFTrDA	72629-94-8	9.17	0.64	8.84	8.53	5.03	8.76	7.65
Perfluorotetradecanoic acid	PFTeDA	376-06-7	9.80	1.03	9.12	8.78	5.30	9.42	7.90
Perfluoropentadecanoic acid	PFPeDA	141074-63-7	10.48	1.42	9.43	9.06	5.68	10.14	8.21
Perfluorohexadecanoic acid	PFHxDA	67905-19-5	11.16	1.92	9.64	9.24	5.86	10.82	8.37
Perfluoroheptadecanoic acid	PFHpDA	57475-95-3	11.72	2.32	9.81	9.40	6.31	11.42	8.56
Perfluorooctadecanoic acid	PFODA	16517-11-6	12.53	2.69	10.29	9.84	6.62	12.25	9.00
Perfluorononadecanoic acid	PFNDA	133921-38-7	12.95	3.28	10.12	9.67	6.81	12.65	8.79
Perfluoroeicosanoic acid	PFEiA	68310-12-3	13.85	3.73	10.64	10.12	7.08	13.61	9.29
2-(Perfluorobutyl)ethanoic acid	4:2 FTCA		3.12	-3.25	6.31	6.37	3.07	2.67	5.52
2-(Perfluorohexyl)ethanoic acid	6:2 FTCA	53826-12-3	4.54	-2.39	6.93	6.93	3.70	4.14	6.09
2-(Perfluorooctyl)ethanoic acid	8:2 FTCA	27854-31-5	5.88	-1.63	7.58	7.51	4.41	5.52	6.69
2-(Perfluorodecyl)ethanoic acid	10:2 FTCA	53826-13-4	7.19	-0.81	8.13	8.01	5.02	6.85	7.17
2H,2H,3H,3H-Perfluorohexanoic acid	3:3 FTCA	356-02-5	2.76	-3.50	6.21	6.25	3.29	2.27	5.36
2H,2H,3H,3H-Perfluorooctanoic acid	5:3 FTCA	914637-49-3	4.08	-2.75	6.84	6.83	3.96	3.62	5.94

2H,2H,3H,3H-Perfluorodecanoic acid	7:3 FTCA	812-70-4	5.50	-1.81	7.38	7.31	4.55	5.09	6.44
(Z)-2H-Perfluoro-2-hexenoic acid	Z-4:2 FTUCA		3.31	-2.68	5.97	5.98	2.97	2.79	5.08
(E)-2H-Perfluoro-2-hexenoic acid	E-4:2 FTUCA		3.32	-2.69	6.02	6.01	2.87	2.84	5.17
(Z)-2H-Perfluoro-2-octenoic acid	Z-6:2 FTUCA	70887-88-6 (racemic)	4.68	-1.86	6.58	6.54	3.60	4.20	5.65
(E)-2H-Perfluoro-2-octenoic acid	E-6:2 FTUCA	70887-88-6 (racemic)	4.65	-1.97	6.66	6.61	3.53	4.20	5.76
(Z)-2H-Perfluoro-2-decenoic acid	Z-8:2 FTUCA	70887-84-2 (racemic)	6.04	-0.99	7.14	7.03	4.18	5.60	6.15
(E)-2H-Perfluoro-2-decenoic acid	E-8:2 FTUCA	70887-84-2 (racemic)	6.08	-1.05	7.25	7.13	4.20	5.67	6.30
(Z)-2H-Perfluoro-2-dodecenoic acid	Z-10:2 FTUCA	70887-94-4 (racemic)	7.39	-0.26	7.81	7.66	4.92	6.99	6.78
(E)-2H-Perfluoro-2-dodecenoic acid	E-10:2 FTUCA	70887-94-4 (racemic)	7.40	-0.34	7.92	7.74	4.92	7.03	6.91
Perfluorooctane sulfonamidoacetic acid	FOSAA	2806-24-8	5.84	-5.41	11.20	11.24	6.12	5.09	9.80
N-Methylperfluorooctane sulfonamidoacetic acid	MeFOSAA	2355-31-9	6.24	-3.85	10.14	10.09	6.56	5.99	9.21
N-Ethylperfluorooctane sulfonamidoacetic acid	EtFOSAA	2991-50-6	6.63	-3.98	10.68	10.61	7.19	6.38	9.70
2,3,3,3-Tetrafluoro-2-(1,1,2,2,3,3,3-heptafluoropropoxy)propanoic acid	HFPO-DA	13252-13-6	4.83	-1.43	6.39	6.26	2.38	4.34	5.41
4,8-Dioxa-3H-perfluorononanoic acid	ADONA	919005-14-4	5.44	-1.45	6.98	6.88	3.02	5.08	6.12
Perfluoro(2-ethoxyethane)sulfonic acid	PFEESA	113507-82-7	4.48	-3.49	8.06	7.97	3.34	3.73	6.77
Perfluoro-3,6-dioxaheptanoic acid	NFDHA	151772-58-6	4.62	-1.81	6.54	6.43	2.58	4.14	5.57
Perfluoro-3-methoxypropanoic acid	PFMPA	377-73-1	3.40	-2.67	6.14	6.07	2.16	2.89	5.21
Perfluoro(4-methoxybutanoic) acid	PFMBA	863090-89-5	4.09	-2.28	6.46	6.37	2.51	3.59	5.50
Trifluoromethanesulfonic acid	TFSA	1493-13-6	1.92	-5.53	7.45	7.45	2.75	1.15	6.27
Pentafluoroethanesulfonic acid	PFES	354-88-1	2.69	-4.98	7.68	7.67	2.98	1.88	6.43
Perfluoropropanesulfonic acid	PFPrS	423-41-6	3.38	-4.65	8.07	8.03	3.41	2.58	6.79
Perfluorobutanesulfonic acid	PFBS	375-73-5	4.09	-4.21	8.38	8.30	3.77	3.30	7.06
Perfluoropentanesulfonic acid	PFPeS	2706-91-4	4.82	-3.82	8.75	8.64	4.05	4.05	7.41
Perfluorohexanesulfonic acid	PFHxS	355-46-4	5.49	-3.36	8.98	8.85	4.31	4.73	7.62
Perfluoroheptanesulfonic acid	PFHpS	375-92-8	6.14	-3.02	9.32	9.16	4.72	5.37	7.90
Perfluorooctanesulfonic acid	PFOS	1763-23-1	6.84	-2.73	9.76	9.57	5.10	6.11	8.33
Perfluorononanesulfonic acid	PFNS	68259-12-1	7.53	-2.18	9.94	9.71	5.31	6.83	8.50
Perfluorodecanesulfonic acid	PFDS	335-77-3	8.27	-1.83	10.36	10.10	5.67	7.59	8.88
Perfluoro-4-ethylcyclohexanesulfonic acid	PFECHS	646-83-3	5.78	-4.10	10.03	9.88	5.32	4.96	8.54
2-(Perfluorobutyl)ethane-1-sulfonic acid	4:2 FTS	757124-72-4	3.04	-6.57	9.46	9.61	5.02	2.63	8.61

2-(Perfluorohexyl)ethane-1-sulfonic acid	6:2 FTS	27619-97-2	4.41	-5.66	9.98	10.07	5.59	4.03	9.07
2-(Perfluorooctyl)ethane-1-sulfonic acid	8:2 FTS	39108-34-4	5.75	-4.85	10.56	10.60	6.17	5.42	9.62
2-(Perfluorodecyl)ethane-1-sulfonic acid	10:2 FTS	120226-60-0	7.14	-4.05	11.21	11.19	6.88	6.82	10.19
9-Chlorohexadecafluoro-3-oxanonane-1-sulfonic acid	9Cl-PF3ONS	756426-58-1	7.42	-2.45	10.09	9.87	5.39	6.74	8.65
11-Chloroeicoafluoro-3-oxaundecane-1-sulfonic acid	11Cl-PF3OUdS	83329-89-9	8.91	-1.58	10.77	10.49	6.20	8.25	9.26
Mono[2-(perfluorohexyl)ethyl] hydrogen phosphate	6:2 monoPAP	57678-01-0	4.19	-9.96	13.85	14.15	6.15	2.36	11.45
Mono[2-(perfluorooctyl)ethyl] hydrogen phosphate	8:2 monoPAP	57678-03-2	5.65	-9.62	15.02	15.27	6.94	3.66	12.36
Bis[2-(perfluorohexyl)ethyl] hydrogen phosphate	6:2 diPAP	57677-95-9	9.44	-3.36	12.84	12.80	8.05	8.54	11.04
(Perfluorohexyl)ethyl (perfluorooctyl)ethyl hydrogen phosphate	6:2/8:2 diPAP	943913-15-3	10.92	-2.66	13.69	13.57	9.19	10.12	11.86
Bis[2-(perfluorooctyl)ethyl] hydrogen phosphate	8:2 diPAP	678-41-1	11.86	-1.40	13.42	13.26	8.88	11.15	11.65
Bis(perfluorohexyl)phosphinic acid	6:6 PFPi	70609-44-8	9.07	-0.70	9.98	9.77	5.39	8.28	8.40
(perfluorooctyl)(perfluorohexyl)phosphinic acid	6:8 PFPi	610800-34-5	10.33	0.07	10.54	10.26	6.00	9.65	8.97
Perfluoro-1-methylheptane sulfonic acid	P1MHpS		6.75	-2.92	9.86	9.67	5.13	5.95	8.36
Perfluoro-3,5,5-trimethylhexanoic acid	P355TMHxA		5.71	-2.07	7.95	7.78	4.07	5.20	6.84
Perfluoro-3,5-dimethylhexanoic acid	P35DMHxA		5.34	-1.98	7.47	7.32	3.56	4.83	6.41
Perfluoro-3,5-dimethylhexane sulfonic acid	P35DMHxS		6.31	-3.38	9.87	9.69	5.21	5.56	8.43
Perfluoro-3,7-dimethyloctanoic acid	P37DMOA		6.66	-1.15	8.02	7.82	4.15	6.19	6.91
Perfluoro-3-methylheptanoic acid	P3MHpA		5.56	-1.62	7.33	7.17	3.42	5.05	6.27
Perfluoro-3-methylheptane sulfonic acid	P3MHpS		6.53	-3.04	9.74	9.56	5.12	5.78	8.31
Perfluoro-4,4-dimethylhexanoic acid	P44DMHxA		5.21	-2.34	7.70	7.55	3.76	4.68	6.61
Perfluoro-4,4-dimethylhexane sulfonic acid	P44DMHxS		6.21	-3.54	9.91	9.75	5.29	5.43	8.46
Perfluoro-4,5-dimethylhexanoic acid	P45DMHxA		5.24	-2.18	7.57	7.42	3.64	4.73	6.51
Perfluoro-4,5-dimethylhexane sulfonic acid	P45DMHxS		6.26	-3.41	9.83	9.68	5.27	5.46	8.37
Perfluoro-4-methylheptanoic acid	P4MHpA		5.51	-1.89	7.55	7.39	3.59	5.00	6.48
Perfluoro-4-methylheptane sulfonic acid	P4MHpS		6.56	-2.98	9.72	9.54	5.08	5.81	8.29
Perfluoro-5,5-dimethylhexanoic acid	P55DMHxA		5.32	-2.23	7.69	7.55	3.73	4.79	6.60
Perfluoro-5,5-dimethylhexane sulfonic acid	P55DMHxS		6.35	-3.25	9.77	9.60	5.18	5.59	8.33
Perfluoro-5-methylheptanoic acid	P5MHpA		5.53	-1.84	7.54	7.38	3.59	5.03	6.47
Perfluoro-5-methylheptane sulfonic acid	P5MHpS		6.55	-3.02	9.75	9.57	5.18	5.77	8.29
Perfluoro-6-methylheptanoic acid	P6MHpA		5.65	-1.74	7.55	7.39	3.64	5.16	6.50
Perfluoro-6-methylheptane sulfonic acid	P6MHpS		6.66	-3.00	9.84	9.66	5.15	5.90	8.38
Perfluoro-7-methyloctanoic acid	ipPFNA		6.30	-1.33	7.82	7.63	3.88	5.81	6.72
Perfluoro-7-methyloctane sulfonic acid	ipPFNS		7.34	-2.55	10.11	9.89	5.43	6.64	8.66

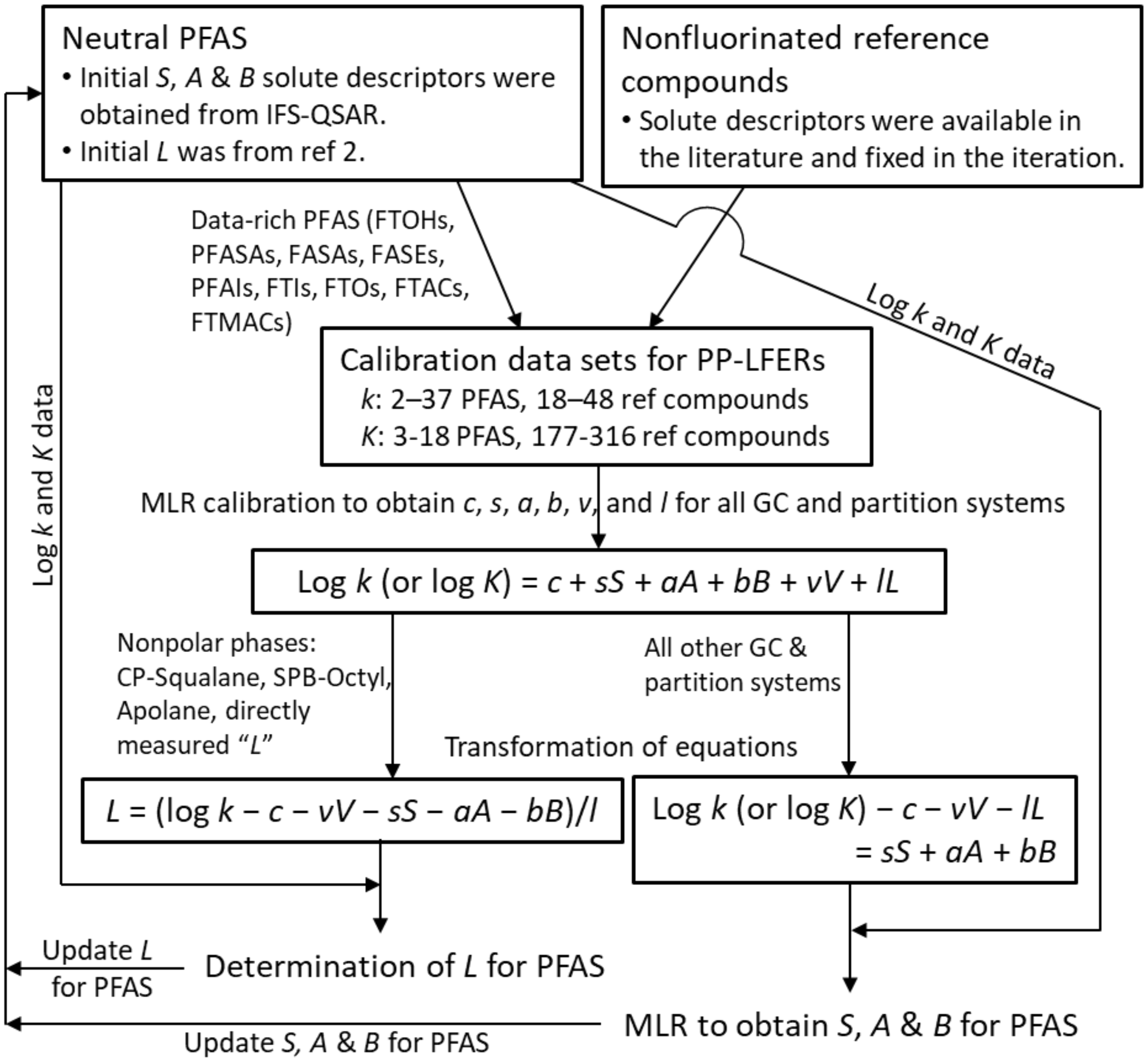

Figure S1. Schematic illustration of the iterative calculation procedure for PFAS solute descriptors and system parameters.

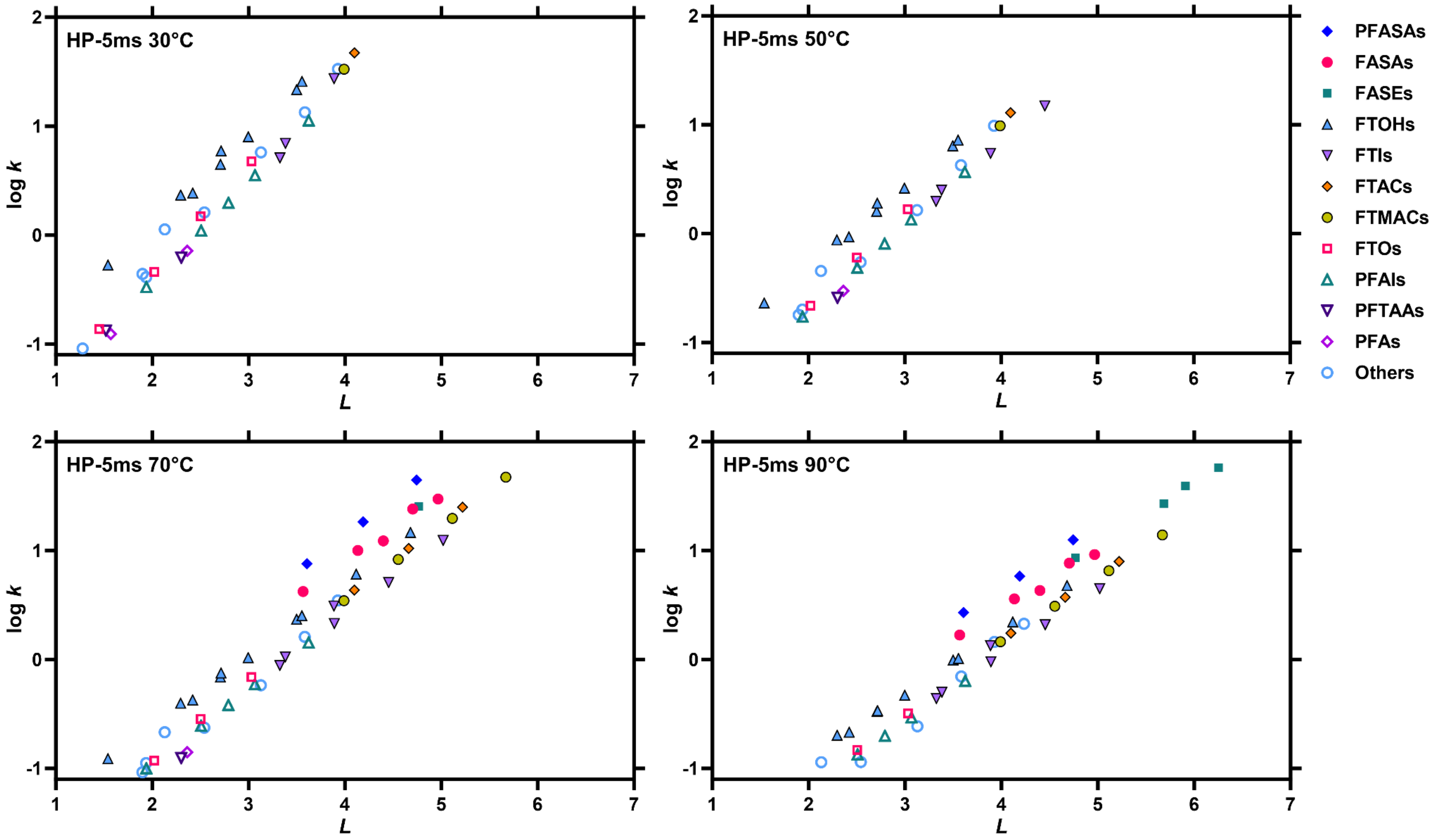

Figure S2. Measured log *k* with HP-5ms vs log $K_{Hxd/air}$ (*L*).

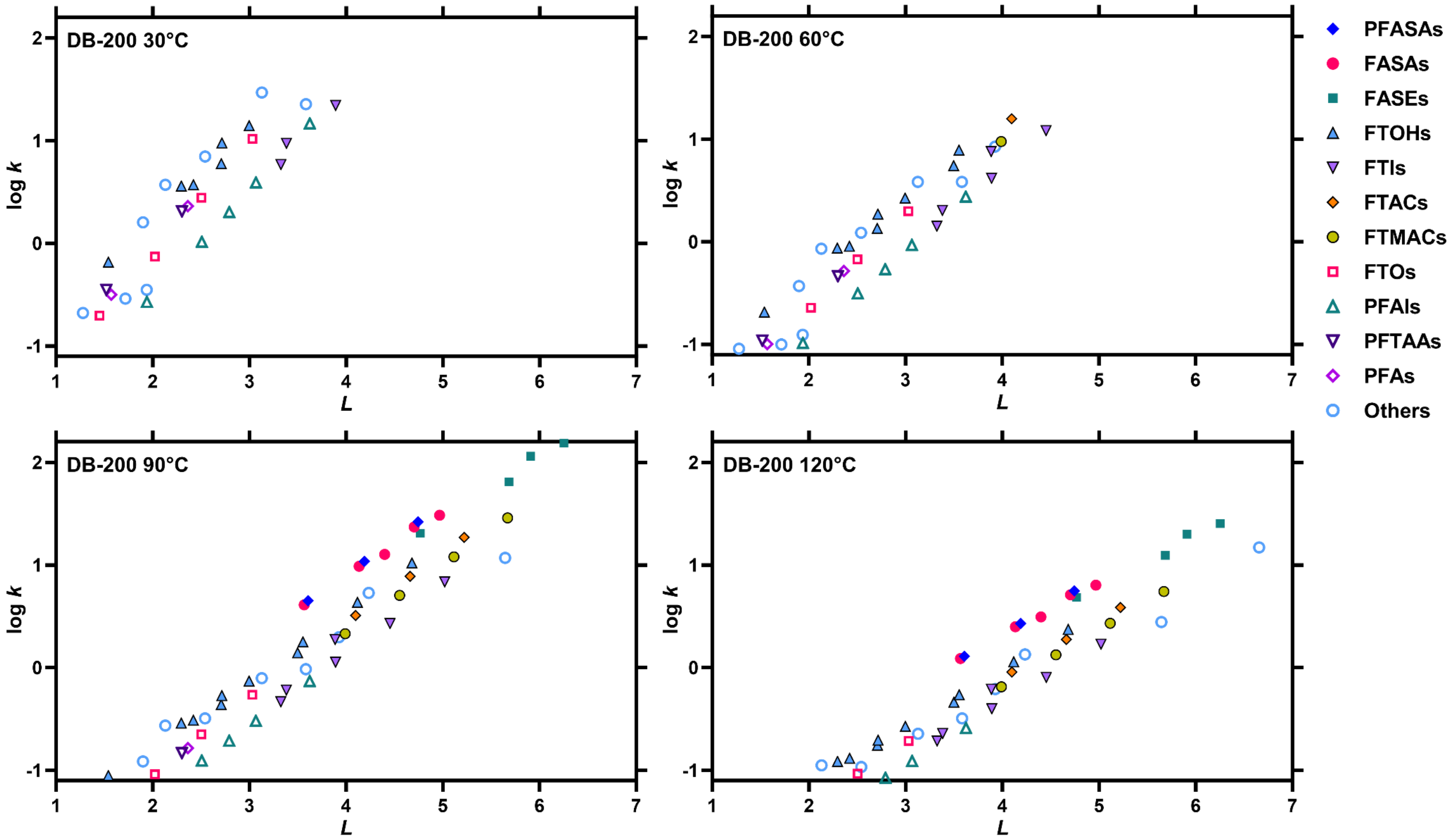

Figure S3. Measured log *k* with DB-200 vs log $K_{Hxd/air}$ (*L*).

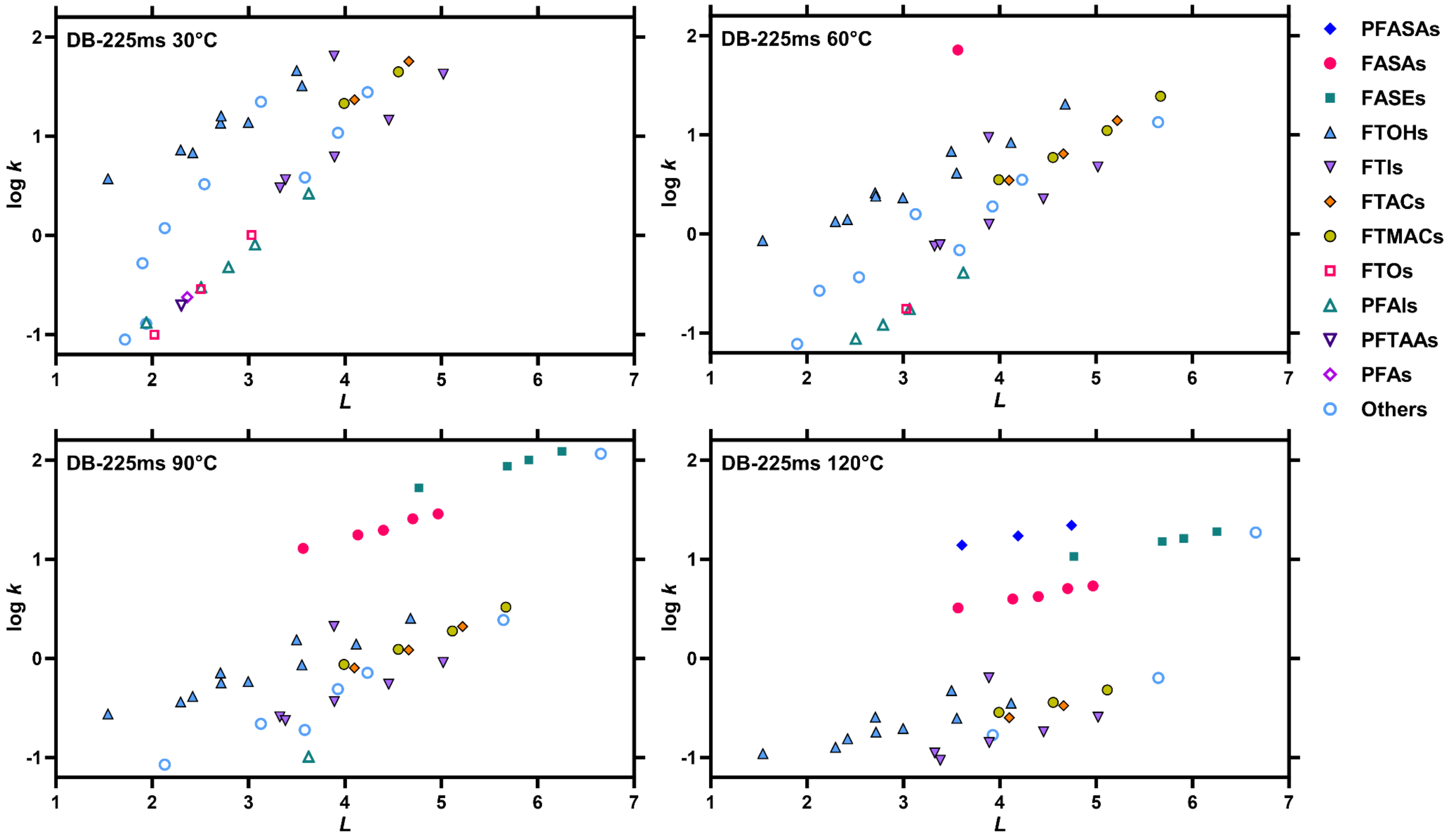

Figure S4. Measured log *k* with DB-225ms vs log $K_{Hxd/air}$ (*L*).

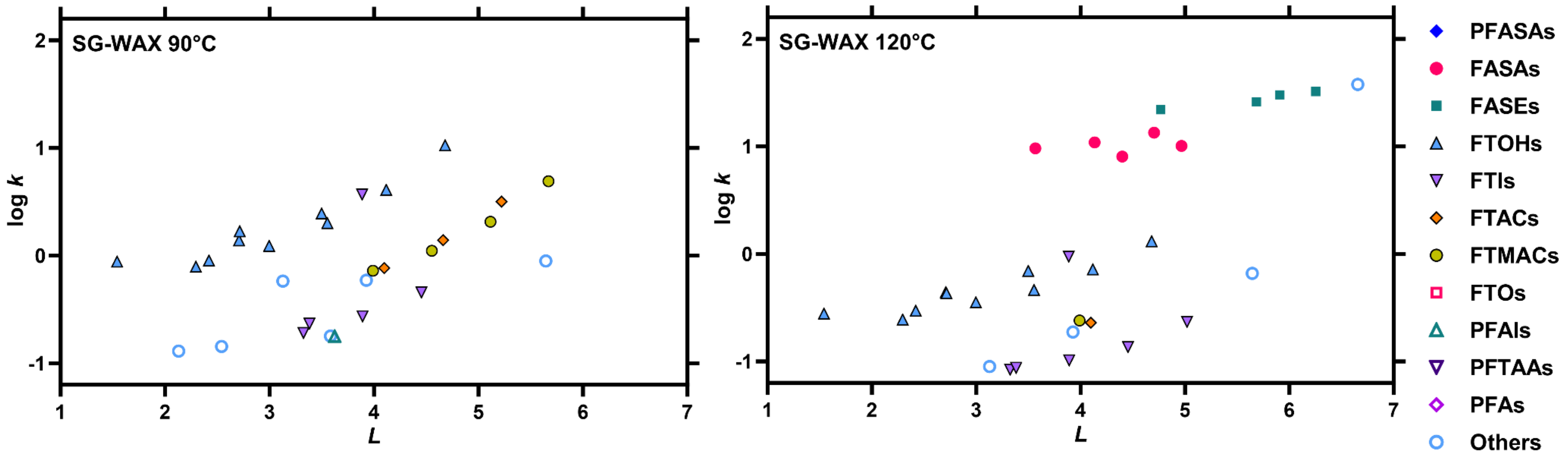

Figure S5. Measured log *k* with SolGel-WAX vs log $K_{Hxd/air}$ (L).

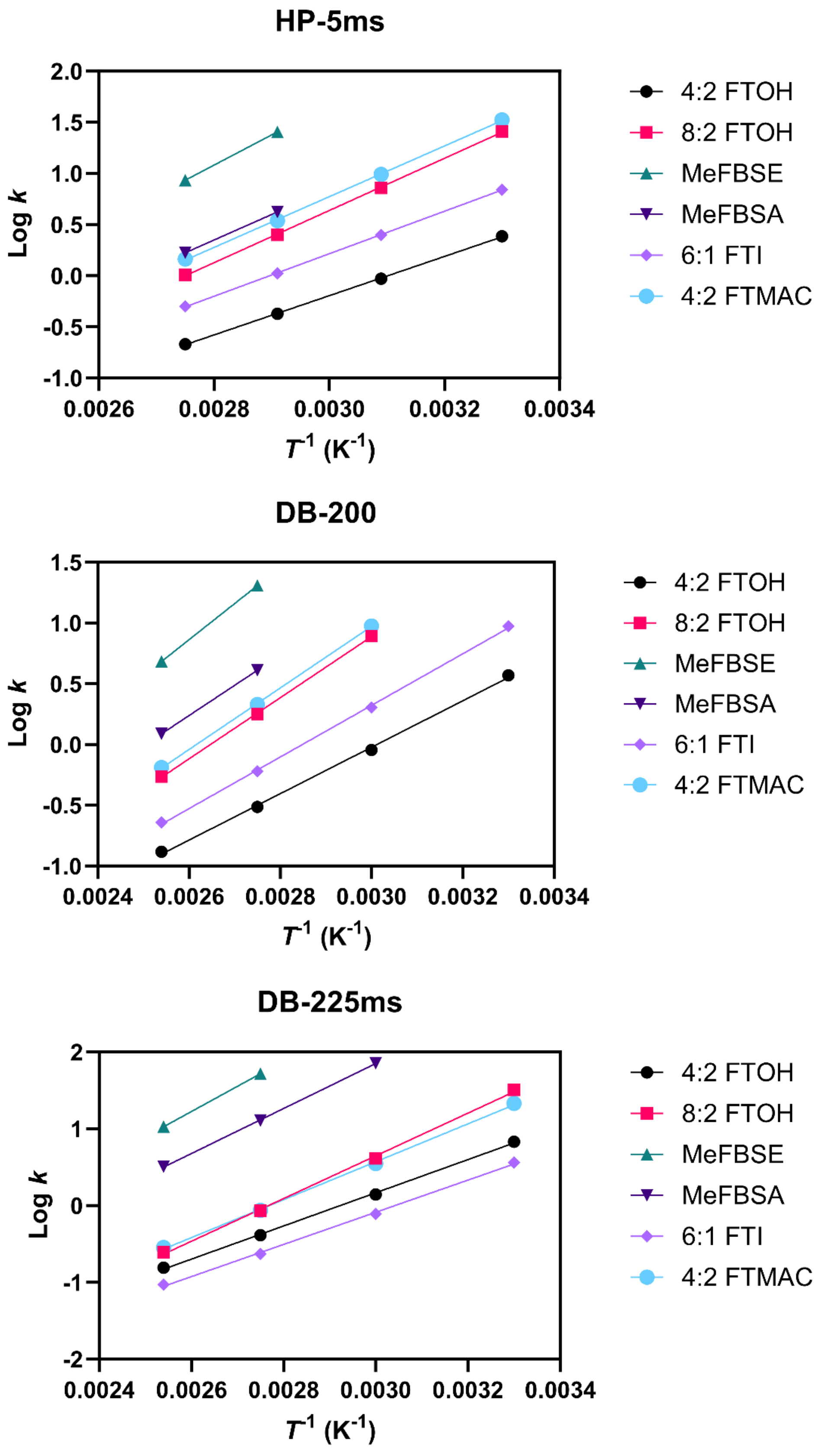

Figure S6. Log *k* against the reciprocal of temperature for selected PFAS.

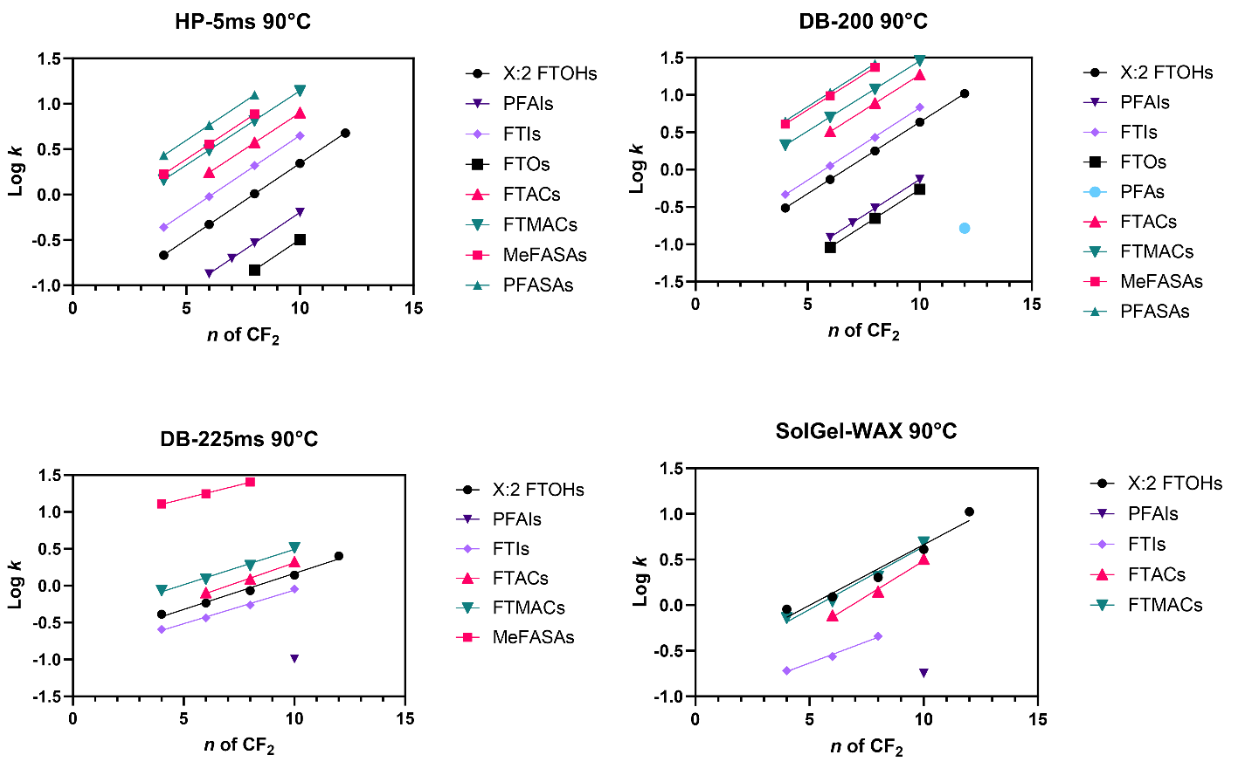

Figure S7. Log *k* against the number of CF_2 units for selected groups of PFAS.

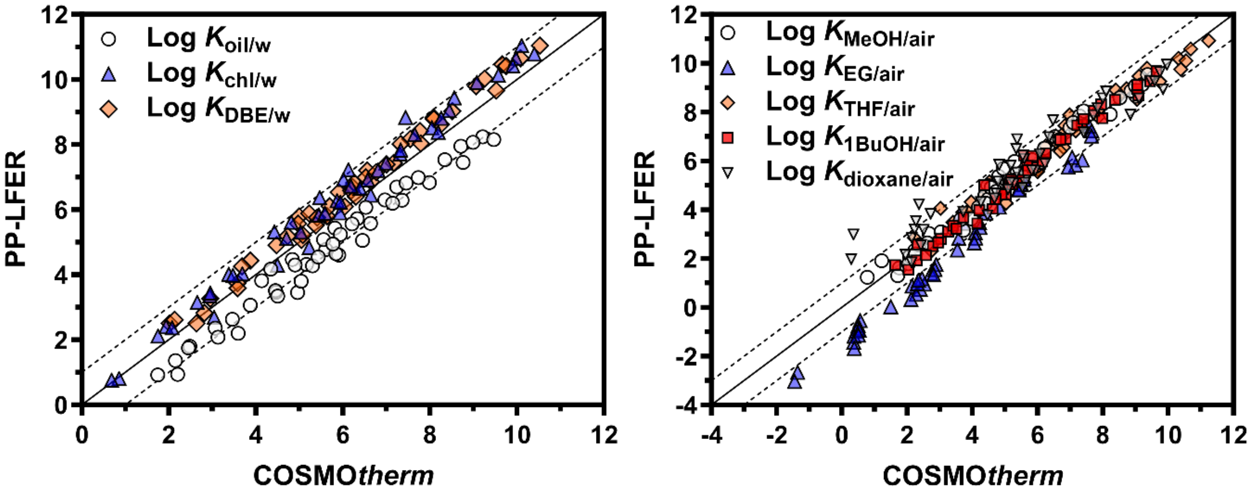

Figure S8. Predictions from PP-LFERs vs COSMO*therm* for 47 PFAS. The PP-LFER equations used to predict chloroform/water ($K_{chl/w}$) and dibutyl ether/water ($K_{DBE/w}$) partition coefficients are from ref 3, and those used to predict methanol/air ($K_{MeOH/w}$), ethylene glycol/air ($K_{EG/air}$), tetrahydrofuran/air ($K_{THF/air}$), 1-butanol/air ($K_{1BuOH/air}$), and 1,4-dioxane/air ($K_{dioxane/air}$) partition coefficients are from ref 4. The PP-LFER equation for oil/water partition coefficients ($K_{oil/w}$) was recalibrated using data from refs 5 and 6 with the solute descriptors for FTOHs updated in this study. All solvents were in the "dry" state (i.e., without water) in the COSMO*therm* calculation. Trinonanoylglycerol was used as the model oil for COSMO*therm*, following the approach in ref 7. The solid lines indicate the 1:1 agreement and the dashed lines the ± 1 log unit deviation.

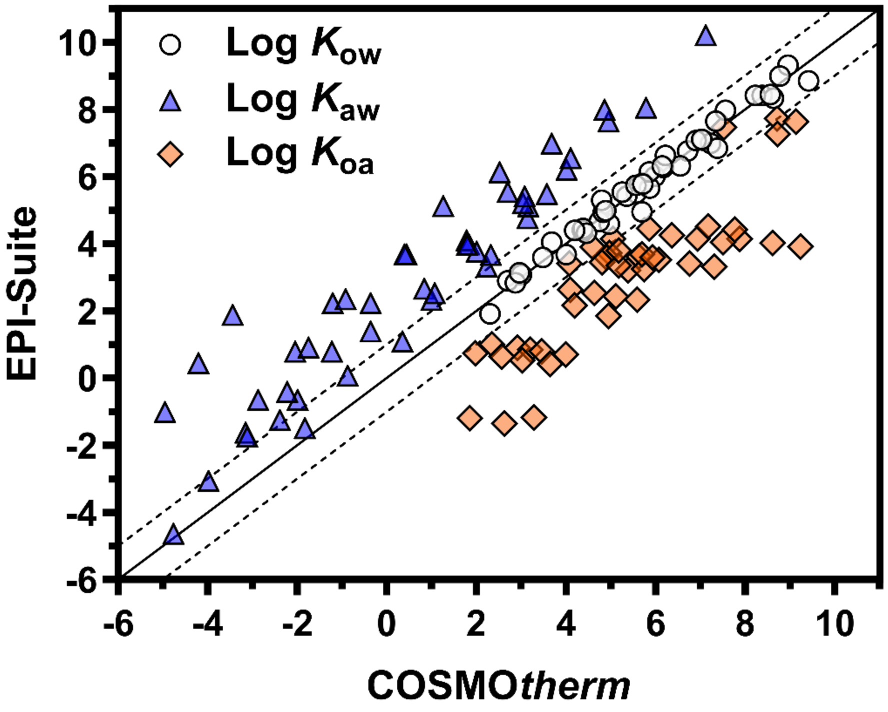

Figure S9. Partition coefficients predicted by EPI-Suite and COSMO*therm*. The solid lines indicate the 1:1 agreement and the dashed lines the ± 1 log unit deviation.

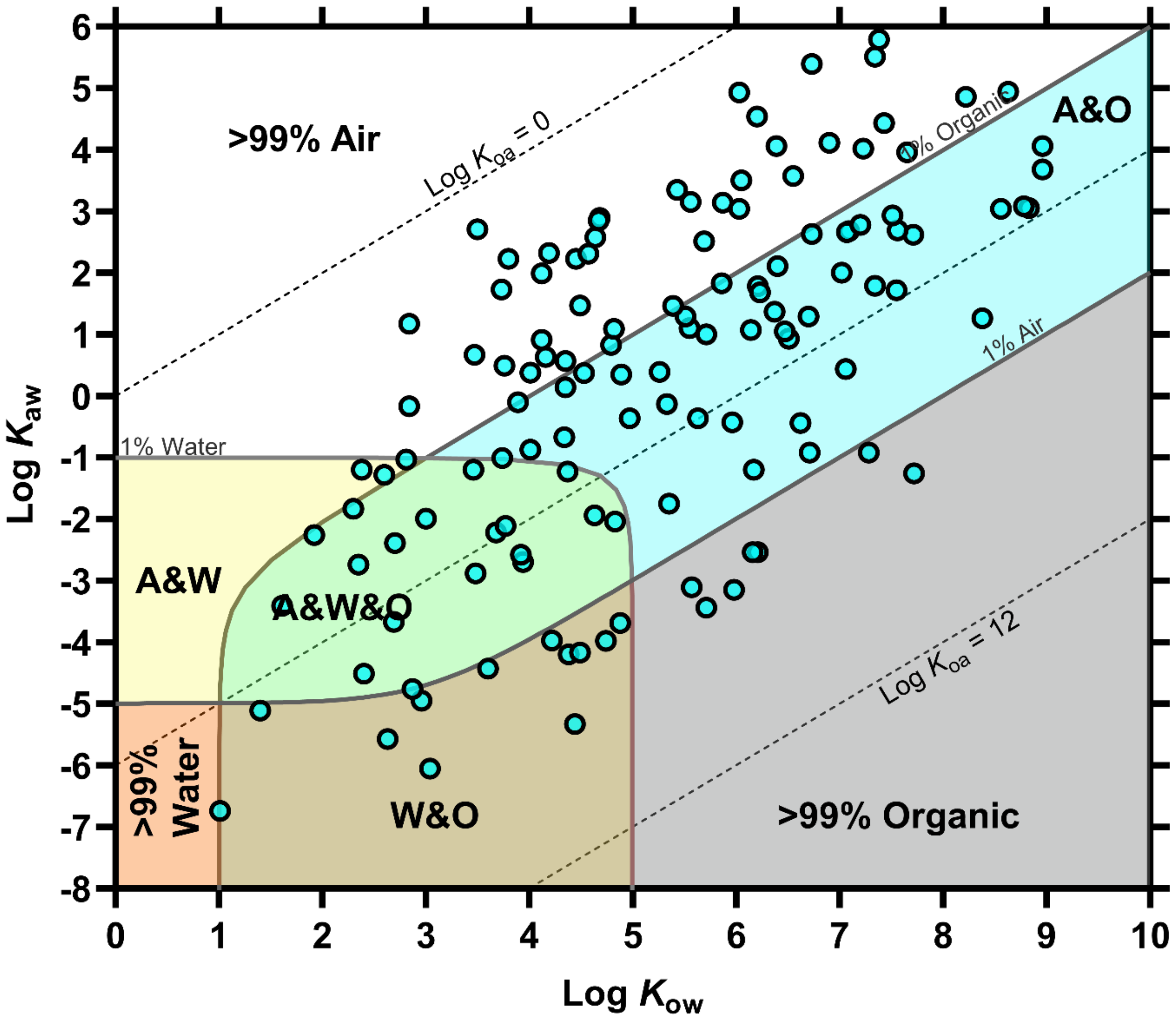

Figure S10. Chemical partitioning space plot for 134 neutral PFAS using COSMO*therm*-predicted partition coefficients.

References

1. Endo, S.; Hammer, J.; Matsuzawa, S. Experimental determination of air/water partition coefficients for 21 per- and polyfluoroalkyl substances reveals variable performance of property prediction models. *Environ. Sci. Technol.* **2023,** *57* (22), 8406-8413.
2. Hammer, J.; Endo, S. Volatility and nonspecific van der Waals interaction properties of per- and polyfluoroalkyl substances (PFAS): Evaluation using hexadecane/air partition coefficients. *Environ. Sci. Technol.* **2022,** *56* (22), 15737-15745.
3. Goss, K.-U. Predicting the equilibrium partitioning of organic compounds using just one linear solvation energy relationship (LSER). *Fluid Phase Equilib.* **2005,** *233* (1), 19-22.
4. Flanagan, K. B.; Acree, W. E., Jr.; Abraham, M. H. Comments regarding "predicting the equilibrium partitioning of organic compounds using just one linear solvation energy relationship (LSER)". *Fluid Phase Equilib.* **2005,** *237* (1-2), 224-226.
5. Geisler, A.; Endo, S.; Goss, K.-U. Partitioning of organic chemicals to storage lipids: Elucidating the dependence on fatty acid composition and temperature. *Environ. Sci. Technol.* **2012,** *46* (17), 9519-9524.
6. Endo, S.; Goss, K.-U. Predicting partition coefficients of polyfluorinated and organosilicon compounds using polyparameter linear free energy relationships (PP-LFERs). *Environ. Sci. Technol.* **2014,** *48* (5), 2776-2784.
7. Geisler, A.; Oemisch, L.; Endo, S.; Goss, K.-U. Predicting storage–lipid water partitioning of organic solutes from molecular structure. *Environ. Sci. Technol.* **2015,** *49* (9), 5538-5545.
